# Differentiated Mechanisms of Biochar Mitigating Straw-Induced Greenhouse Gas Emissions in Two Contrasting Paddy Soils

**DOI:** 10.3389/fmicb.2018.02566

**Published:** 2018-11-13

**Authors:** Ya-Qi Wang, Ren Bai, Hong J. Di, Liu-Ying Mo, Bing Han, Li-Mei Zhang, Ji-Zheng He

**Affiliations:** ^1^State Key Laboratory of Urban and Regional Ecology, Research Center for Eco-Environmental Sciences, Chinese Academy of Sciences, Beijing, China; ^2^University of Chinese Academy of Sciences, Beijing, China; ^3^Centre for Soil and Environmental Research, Lincoln University, Lincoln, New Zealand; ^4^Beihai Forestry Research Institute, Beihai, China

**Keywords:** paddy soil, biochar, straw return, CH_4_, N_2_O, functional genes

## Abstract

Straw returns to the soil is an effective way to improve soil organic carbon and reduce air pollution by straw burning, but this may increase CH_4_ and N_2_O emissions risks in paddy soils. Biochar has been used as a soil amendment to improve soil fertility and mitigate CH_4_ and N_2_O emissions. However, little is known about their interactive effect on CH_4_ and N_2_O emissions and the underlying microbial mechanisms. In this study, a 2-year pot experiment was conducted on two paddy soil types (an acidic Utisol, TY, and an alkaline Inceptisol, BH) to evaluate the influence of straw and biochar applications on CH_4_ and N_2_O emissions, and on related microbial functional genes. Results showed that straw addition markedly increased the cumulative CH_4_ emissions in both soils by 4.7- to 9.1-fold and 23.8- to 72.4-fold at low (S1) and high (S2) straw input rate, respectively, and significantly increased *mcrA* gene abundance. Biochar amendment under the high straw input (BS2) significantly decreased CH_4_ emissions by more than 50% in both soils, and increased both *mcrA* gene and *pmoA* gene abundances, with greatly enhanced *pmoA* gene and a decreased *mcrA*/*pmoA* gene ratio. Moreover, methanotrophs community changed distinctly in response to straw and biochar amendment in the alkaline BH soil, but showed slight change in the acidic TY soil. Straw had little effect on N_2_O emissions at low input rate (S1) but significantly increased N_2_O emissions at the high input rate (S2). Biochar amendment showed inconsistent effect on N_2_O emissions, with a decreasing trend in the BH soil but an increasing trend in the TY soil in which high ammonia existed. Correspondingly, increased *nirS* and *nosZ* gene abundances and obvious community changes in *nosZ* gene containing denitrifiers in response to biochar amendment were observed in the BH soil but not in the TY soil. Overall, our results suggested that biochar amendment could markedly mitigate the CH_4_ and N_2_O emissions risks under a straw return practice via regulating functional microbes and soil physicochemical properties, while the performance of this practice will vary depending on soil parent material characteristics.

## Introduction

Global warming caused by the continued increase in anthropogenic greenhouse gas (GHG) emissions is expected to exert a severe impact on the stability of natural ecosystems and sustainable development of human society (Smith and Fang, 2010). The mitigation of GHG emissions remains a formidable challenge in the quest to slow climate change. The respective global warming potentials of methane (CH_4_) and nitrous oxide (N_2_O) are 23- and 298-fold higher than that of carbon dioxide (Munoz et al., 2010), and contribute around 17 and 6% to radiative forcing, respectively (WMO, 2017). Paddy soil is one of the important sources of atmospheric CH_4_ and N_2_O, with average annual emissions of 7.22–8.64 Tg and 88.0–98.1 Gg in China, respectively (Xing, 1998; Li et al., 2004; Liu et al., 2010). A strategy to mitigate CH_4_ and N_2_O emissions in rice paddies is imperative.

Crop residues have been widely applied in agriculture as a source of nutrients to improve soil fertility, and showed significant effects on improving soil organic C stocks (Li et al., 2010), reducing environmental pollution associated with straw burning (Kharub et al., 2004; Romasanta et al., 2017), and regulating carbon and nitrogen cycling (Lugato et al., 2006). However, the application of crop residues can increase the production of atmospheric GHGs (Zou et al., 2005; Ma et al., 2008; Hang et al., 2014). For example, the global warming potential was significantly enhanced by straw incorporation from a rice paddy field, with CH_4_ increase by 3–11 times in straw-contained soils compared to the control (Ma et al., 2007). Therefore, it is crucial to find a method to mitigate the emissions of GHGs induced by straw application in rice paddy fields.

Biochar, a carbon sequestrating and recalcitrant material, is produced by the pyrolysis of plant residues under a zero or limited oxygen condition (Cao et al., 2011), possessing the characteristics of a high pH, high cation exchange capacity (CEC), and a high hydrophilic characteristic, large porosity and surface area (Lehmann et al., 2011). Biochar has been applied to soil as an optional amendment to improve soil fertility and grain yields via the promotion of nutrient turnover (Zhang et al., 2010; Kang et al., 2016). Soil with a low fertility or pH value can be improved with biochar amendment (Bakar et al., 2015). Biochar amendment can also regulate CH_4_ and N_2_O emissions from rice paddy soils (He et al., 2017). For example, CH_4_ emissions were suppressed by 39.5% by adding biochar to a paddy soil under elevated temperature and CO_2_ (Han et al., 2016). However, another study found that N_2_O emissions significantly decreased following biochar addition, while CH_4_ emissions increased, probably resulting from an improvement in microbial growth due to the supply of additional C (Singla and Inubushi, 2014). Either no effect (Brassard et al., 2016) or stimulation (Yu et al., 2013) of biochar-induced GHG emissions have also been observed, illustrating an apparent dependence on biochar and soil properties (Singla and Inubushi, 2014). However, the mechanism remains unclear as to how the soil interacts with biochar with respect to CH_4_ and N_2_O emissions.

CH_4_ and N_2_O emissions in paddy soils reflect the balance of production and consumption processes which are associated with microbial activities in soil (Yan et al., 2000; Bodelier, 2015). For example, soil organic matter decomposed by various microorganisms is ultimately utilized by the methanogenic archaea with the production of CH_4_, which can be consumed by the methanotrophic proteobacteria as a sole source of carbon and energy before release to the atmosphere (Bridgham et al., 2013). N_2_O emission from soil is also dependent on the balance of N_2_O reduction and production processes and is influenced by multiple factors. However, there are no consistent conclusions on the influence of biochar amendment on soil microbial communities involved in CH_4_ and N_2_O production and consumption. Improved abundance of N_2_O-reducing bacteria has been observed after biochar amendment, promoting the reduction of N_2_O to N_2_ during denitrification thus decreasing N_2_O emissions (Harter et al., 2014). Similarly, several other studies reported that biochar amendment reduced N_2_O emissions by increasing nitrous oxide reductase encoding gene (*nosZ*) due to soil pH increases (Van Zwieten et al., 2014; Xu et al., 2014). Conversely, increased N_2_O emissions stimulated by biochar amendment in a rice paddy soil was found to be correlated with the increased bacterial ammonia monooxygenase encoding *amoA* gene, but not with nitrous oxide reductase encoding gene (*nosZ*) and nitrite reductase encoding genes (*nirK* and *nirS*) (Lin et al., 2017).

Among the environmental and edaphic factors influencing the microbial processes, the soil C/N ratio plays a pivotal role in controlling the shifts among key functional microbial processes with separate redox conditions (Kraft et al., 2014). Remarkably, a higher C/N ratio would favor anammox or dissimilatory nitrate reduction to ammonium (DNRA) while a lower ratio would contribute to denitrification (Tiedje et al., 1982; Kraft et al., 2014; Shan et al., 2016). Furthermore, the soil redox potential (*Eh*) and pH are also essential factors largely deciding the availabilities of electron transfer for microbial-mediated processes and microbial metabolism (Kralova et al., 1992; DeAngelis et al., 2010). Otherwise, soil carbon dynamics are highly relevant with the growth of microorganisms involved in GHGs (Wang et al., 2017). Therefore, the amount of straw addition could regulate GHGs emissions not only via influencing the availability of soil organic C (Wu et al., 2013; Liu et al., 2014; Zhang et al., 2014; Zhong et al., 2017), but also via adjusting the soil C/N ratio, and different amounts of straw returns exerted different effects on soil microbial activities and GHGs emissions (Naser et al., 2007). Furthermore, individual rather than interactive effects of straw and biochar amendments on CH_4_ and N_2_O emissions were the focus of earlier studies (Shen et al., 2014; Ly et al., 2015; Thammasom et al., 2016). Consequently, more studies are required to estimate the influence of biochar amendment on CH_4_ and N_2_O emissions under different rates of straw incorporation, and the processes controlling the gaseous emissions should be identified. Therefore an experiment involving straw and biochar amendments was conducted in two types of paddy soils to evaluate the dynamics of CH_4_ and N_2_O emissions in this study. Two rice straw levels were applied to construct different soil C/N ratio, and the effects of biochar on CH_4_ and N_2_O emissions were monitored. Microbial functional genes involved in the production and consumption of CH_4_ and N_2_O were analyzed. The specific objectives were to: (1) Evaluate the effects of the biochar addition on the CH_4_ and N_2_O emissions in rice paddy soils under different rates of straw incorporation; (2) Quantify the responses of different functional microbial groups to biochar and straw amendments under two contrasting soil types and evaluate whether the difference in microbial groups might explain the variation in CH_4_ and N_2_O formation and release from the soils.

## Materials and Methods

### Soil Information and Pot Experiment Setup

The paddy soils were originally collected from Taoyuan (TY, 111.48° E, 28.90° N), Hunan Province, and Binhai (BH, 119.84° E, 34.01° N), Jiangsu Province, rice production areas in Southeast China. The soils were classified as an Inceptisol and an Utisol, respectively, according to the USDA Taxonomy. Fresh soils were air dried to 30–40% maximum field capacity and then passed through a 2 mm sieve, followed by a homogenous mixing before being used for the pot experiment.

The pot experiment was located outdoors in a farm field which received natural day light and ambient temperature in the suburb of Beijing. The experimental design involved two rice straw levels with or without biochar addition, i.e., five treatments: (1) S0, no addition of rice straw (control); (2) S1, 0.33% (w:w) rice straw addition (equal to all aboveground biomass return); (3) S2, 0.66% (w:w) rice straw addition; (4) BS1, 0.33% (w:w) rice straw addition plus 2.0% (w:w) biochar (equal to 45 t ha^−1^); and (5) BS2, 0.66% (w:w) rice straw addition plus 2.0% (w:w) biochar. The rice straw used in the experiment was collected from the area where soil samples were collected, and ground into a powder before use. Biochar was pyrolytically produced from maize straw feedstock under 450°C, and was purchased as a commercial product from Liao Ning Golden Future Agriculture Technology Co., Ltd., with a pH of 9.2, and total carbon, nitrogen, and phosphorus of 679, 9.4, and 7.8 g kg^−1^, respectively. Three replicate pots (26 cm in diameter and 30 cm in height) were setup for each treatment, and each pot contained 10 kg soil (dry weight). For the rice growing season in 2016, before pots were filled, straw or straw plus biochar were thoroughly mixed with the soil according to the treatment, and phosphorus and potassium were applied as a basal fertilizer mixture for all treatments at 90 and 180 kg ha^−1^ P_2_O_5_ and K_2_O, respectively. All pots were flooded for 10 days and then two rice seedlings were transplanted to each pot at day 10 to avoid seedling burnt. Nitrogen fertilizer (72 kg N ha^−1^ as urea) was dissolved in 200 ml deionized water and applied into surface water of each pot before 1 day rice was transplanted. The remaining urea fertilizer (108 kg N ha^−1^ N) was applied after tillering at day 60. The soils were continuously flooded to a depth of 2.5 cm except for 2 weeks of drainage during tillering (from day 43 to day 58, corresponding to days 33–48 after rice transplanting). After the rice growing season in 2016, pots were preserved *in situ* and covered with tarpaulins to reduce anthropogenic disturbance. In spring 2017, tarpaulins were removed and all pots were flooded for 1 month before rice straw and basal fertilizers were applied into soils. The water and fertilizers regime, rice transplanting and daily management were the same as those in 2016, except that biochar was no longer added.

### Gas Sampling and Measurement

The soil N_2_O and CH_4_ fluxes were measured using the static chamber method during the whole rice growing season at 2- or 3-day intervals from 12 June 2016 to 21 August 2016 and from 8 March 2017 to 19 July 2017. A transparent Plexiglass chamber of 30 or 60 cm in height was affixed by a water-filled groove to the top edge of the soil column to ensure an air-tight system. An electrical fan was attached on the top of the chamber to mix the gas in the headspace. On each sampling day, gas collecting was conducted between 10 and 11 a.m., and gas was collected from each pot at 15 min and 30 min after chamber was sealed. For each time, 30 ml of gas was taken from the chamber using a syringe connected with a three-way valve, and then stored in a glass cylinder for next measurement. Gas samples were measured by using a gas chromatograph (Agilent 7890B, Santa Clara, CA, United States) equipped with a flame ionization detector (FID) and an electron capture detector (μECD), and the gas sample (20 ml) was fed into the GC using a syringe manually. Gas fluxes were calculated using a linear regression analysis.

F=ρ×(P/101.3)×(V/A)×(Δc/Δt)×273/(273+T)

Where: *F* was the flux of N_2_O or CH_4_ (μg N_2_O-N m^−2^ h^−1^ or μg CH_4_-C m^−2^ h^−1^), *ρ* was the density of the trace gas at 0°C and 101.3 KPa (kg m^−3^), *P* was the atmospheric pressure of the experimental site (KPa), *V* was the volume of chamber (m^3^), *A* was the surface area of the chamber, Δ*c*/Δ*t* was the rate of N_2_O or CH_4_ accumulation in the chamber (μg m^−3^ h^−1^), *T* was the chamber mean air temperature in Celsius.

Cumulative N_2_O and CH_4_ emissions (E, kg N ha^−1^ for N_2_O, kg C ha^−1^ for CH_4_) were calculated by the following equation:

$$E=\sum_{i=1}^{n}\left(F_{i}+F_{i+1})/2\times (t_{i+1}-t_{i}\right)\times 24$$

Where: *F* was the gas flux (μg N_2_O-N m^−2^ h^−1^ or μg CH_4_-C m^−2^ h^−1^), *n* was the gross number of gas measurement, *i* was the time of sampling, (*t*_i+1_ − *t*) represented the days between the two conjoint gas measurements.

### Soil Sampling and Physicochemical Analysis

Soil samples were taken after gas sampling at day 18, day 58, and day 120, corresponding to rice seedling, tillering, and heading stages, respectively. A soil core (4 cm in diameter) at a depth interval of 0–5 cm was collected from each pot at each sampling time, the core being 10 cm distant from the rice plant. Finally, three cores in each pot were sampled equidistant along the edge of the soil columns to minimize the disturbance. *In situ Eh* measurements were made at rice-transplanting and before soil sampling days by using a PRN-41 soil *Eh* meter (DKK, TOA, Tokyo, Japan). After a homogenous mixing, soil subsamples were stored at 4°C and −40°C for physicochemical determinations and molecular analyses, respectively.

Soil pH was measured in a soil and water suspension (1:2.5 w/w) using a glass electrode. Soil moisture was measured as loss in weight after oven drying at 105°C to constant weight. ${\text{NH}}_{4}^{+}$ and ${\text{NO}}_{3}^{-}$ were extracted with 1 M KCl solution and determined by using a continuous flow analytical system (AA3, SEAL analytical, Germany). Soil dissolved organic carbon (DOC) was extracted with 0.5 M K_2_SO_4_ and determined by a TOC analyzer (Multi N/C 3100, Analytik Jena, German). Soil total carbon (TC) and total nitrogen (TN) were measured by an Elemental analyzer (Vario EL III-Elementar, Germany).

### DNA Extraction and Quantitative PCR

Total DNA was extracted from 0.3 g freeze-dried soil by using a Power Soil DNA Isolation Kit (Mo Bio, Carlsbad, CA, United States) under the guidance of the manufacturer’s instructions, and the quality of the extracted soil DNA was checked by an agarose gel electrophoresis. All the extracted DNA products were stored at −40°C for the next analysis.

Real-time PCR was conducted on an IQ2 system (Bio-Rad Laboratories Inc., Hercules, CA, United States). The abundances of microbial functional genes related to N_2_O emission (archaeal and bacterial *amoA*, *nirK*, *nirS*, and *nosZ* genes), and methanotrophs *pmoA* gene (methane monooxygenase encoding gene) and methanogens *mcrA* gene (methyl coenzyme M reductase encoding gene) were quantified using a SBYR Green assay with the primer pairs and thermal cycle programs as listed in Supplementary Table 1. The qPCR reactions were executed in a 25 μl mixture containing 12.5 μl SYBR Green Premix Ex Taq (TaKaRa Bio Inc.), 1 μl of each primer for *nirK*, *nirS* and *nosZ* (clade I) genes at 10 μM, 2 μL of each primer for archaeal and bacterial *amoA*, *nosZ* (clade II), *mcrA* and *pmoA* genes at 10 μM, and 2 μl of DNA template (1–20 ng). A negative control without DNA template was also conducted in all the qPCR runs. Melting curves aiming to ensure the reaction specificity were conducted at the end of each PCR run. QPCR results were accepted when melting curve is under a single peak, and the amplification efficiencies were in the range between 86.3% and 110.0% with a *R*^2^ value greater than 0.95. To engender a standard curve for qPCR, the amplifications of target genes were performed with the same primer sets mentioned above, following a cloning sequencing. The plasmids DNA containing the correct insert were extracted, purified and quantified, following a 10-fold dilution series as standards for qPCR. Soil DNA samples, standards and negative controls were all included in triplicates in each run.

### High-Throughput Sequencing Analysis of *pmoA* and *nosZ* Genes

To explore the influence of different treatments on microbial community, all soil samples collected at the seedling stage were subjected to high-throughput sequencing analysis for *pmoA* and *nosZ* I genes, and the soils from S0, S2, and BS2 treatments were selected for survey on the variation of *nosZ* I gene containing community over time. The *pmoA* and *nosZ* I genes were amplified with the primers and PCR conditions listed in Supplementary Table 1 in triplicates. And a unique barcode of 6 bp in length were attached in the forward primer at the 5′ end to distinguish the amplicons from different soil samples. Metabarcoded amplicons were purified and sequenced by Illumina Miseq PE300 (Illumina Inc., San Diego, CA, United States).

The sequencing-read data sets were processed using QIIME 1.90 (Caporaso et al., 2010) standard operation pipeline. The raw data was demultiplexed according to the barcode of each sample. Usearch (version 10.0) program (Edgar, 2013) was used to achieve the mergence between the forward and reverse reads, followed by the trimming barcodes from sequences, demultiplexing and quality filter of sequence. Then, filtering chimera, clustering Operational Taxonomic Unit (OTU) at 97% sequence identity and picking out representative sequences from each OTU (Edgar, 2013) were all operated in the same program. Further, the representative sequences were compared to the public databases, GenBank, by using the National Center for Biotechnology Information (NCBI^1^) BLASTn to guarantee the maximum sequence similarity was a *pmoA* or *nosZ* gene. The annotation for taxonomic information of the methanotrophs and *nosZ* gene containers were conducted based on the Fungenes database^2^ and further confirmed by blasting the representative sequence of each OTU against the NCBI GenBank database. To correct the sampling effort, OTUs resampling were rarefied at minimum number of sequences (5,689 reads for *pmoA* gene and 8,356 reads for *nosZ* gene) per sample for downstream analysis.

### Statistical Analysis

Statistical analyses were conducted with SPSS software (version 19, IMB, Inc., United States). Spearman’s correlation was used to determine the relationships among the N_2_O and CH_4_ emissions, soil properties and abundance of microbial functional genes at different rice growing stages. Repeated measures ANOVA was applied to assess the difference of soil properties and gas emissions in different rice growing stages and treatments. One-way analysis of variance (ANOVA) was performed to test for differences in gas emissions, soil characteristics and abundance of microbial functional genes, while significant difference was defined as *P* < 0.05.

Mothur (Schloss et al., 2009) was operated to analyze the alpha and beta diversity. Beta diversity was characterized by Bray-Curtis dissimilarity matrices based on OTU matrices. Cluster analysis was performed with UPGMA (Unweighted Pair Group Method with Arithmetic Mean) using Bray–Curtis distance measures. To identify the critical parameters driving the community diversity of denitrifier, canonical correlation analysis (CCA) were performed using community ecology vegan package of R software (3.2.4). The envfit function (999 permutations) was used to identify the environmental variables, which significantly contributed to the soil microbial community variance.

#### Nucleotide Sequence Accession Numbers

The representative sequences retrieved in this study were deposited in the GenBank database and assigned accession numbers from MH909699 to MH909751 for *pmoA* gene, from MH909601 to MH909698 for *nosZ* gene.

## Results

### Soil Physicochemical Properties

The TY soil had an initial pH_(H_2_O)_ of 5.7, DOC at 89.07 mg kg^−1^, total N at 2.20 g kg^−1^, while the BH soil had an initial pH_(H_2_O)_ of 7.6, DOC at 33.28 mg kg^−1^, and total N at 1.30 g kg^−1^. During the whole rice growing season in 2016, soils properties were significantly impacted by the straw and biochar amendments. For the TY soil, straw addition (S1 and S2) significantly increased DOC by 2.7–42.4%, but showed no significant impact on the soil C/N ratio over the rice growth stage, in comparison with no straw control (S0) (*P* < 0.05) (Table 1). When compared with straw addition alone (S1 and S2), the TY soil pH significantly increased by 0.5–0.8 unit, DOC by 38.16–40.90% and C/N ratio by 24.07–46.16% with the biochar amendment (BS1 and BS2) at day 18, and similar significant increases of soil pH, DOC, and C/N were also observed at day 58 and day 120 (Table 1, *P* < 0.05). For the BH soil, there was no significant effect on the soil C/N ratio and pH with straw addition alone (S1 and S2), but soil DOC increased by 0.90- to 1.22-fold at day 18 (Table 1), in comparison with S0 treatment. The C/N ratio significantly increased by 24.10–28.87% and TN by 4.17–23.85% with the biochar amendments (BS1 and BS2) at day 18, compared with treatments without the biochar amendment (S1 and S2). Notably, soil pH increased over time in both soils from 6.1 to 7.6 in TY and from 7.7 to 8.7 in BH (Table 1) due to the occasional drainage during the heading stage. Both the rice growing stages and treatments showed significant impacts on soil properties, such as DOC and *Eh*, and there was no significant interaction of the treatments and rice growth stages on the ${\text{NH}}_{4}^{+}$, TC and C/N ratio (Table 2).

**Table 1:** Physiochemical parameters in two paddy soils over rice growth stages in 2016.

Days after straw addition	Treatment	pH	*Eh*-surface (mV)	*Eh*-subsurface (mV)	DOC (mg kg^−1^)	${\text{NH}}_{4}^{+}$ (mg kg^−1^)	${\text{NO}}_{3}^{-}$ (mg kg^−1^)	TN g kg^−1^	C/N
18 days (seedling stage)	TY-S0^a^	6.4 ± 0.1B^b^	12.9 ± 31.4A	75.7 ± 125.7A	61.98 ± 18.33B	49.74 ± 3.74A	0.24 ± 0.15A	2.14 ± 0.08B	9.53 ± 0.13C
	TY-S1	6.1 ± 0.02C	19.3 ± 43.3A	38 ± 85.1A	66.19 ± 5.45B	51.19 ± 4.07A	-	2.18 ± 0.10B	9.64 ± 0.25C
	TY-S2	6.4 ± 0.13B	6.9 ± 59.6A	157 ± 83.4A	88.25 ± 31.81AB	50.6 ± 13.62A	1.47 ± 2.15A	2.2 ± 0.05AB	10.03 ± 0.09C
	TY-BS1	6.9 ± 0.13A	-105.3 ± 40.3B	132 ± 26.6A	93.26 ± 13.39AB	52.91 ± 9.43A	0.85 ± 1.11A	2.14 ± 0.12B	11.96 ± 1.27B
	TY-BS2	6.9 ± 0.07A	-90 ± 40.7B	136 ± 91.9A	121.93 ± 31.02A	45.61 ± 13.14A	0.11 ± 0.16A	2.34 ± 0.05A	14.66 ± 1.44A
	BH-S0	7.9 ± 0.22a	180.8 ± 12.4a	30.3 ± 52.5a	46.10 ± 4.68c	8.71 ± 6.88a	9.93 ± 5.65a	1.11 ± 0.02c	19.43 ± 0.36b
	BH-S1	7.8 ± 0.11a	3.8 ± 32.7b	-224.7 ± 140.3b	87.48 ± 12.78abc	12.75 ± 3.02a	0.84 ± 0.53b	1.44 ± 0.14ab	17.99 ± 0.26b
	BH-S2	7.7 ± 0.05a	-6.1 ± 38.3b	-169.7 ± 67.6b	102.53 ± 33.93ab	6.93 ± 4.69a	0.32 ± 0.34b	1.3 ± .15bc	18.8 ± 0.32b
	BH-BS1	7.8 ± 0.06a	-32.1 ± 12.9b	-132.7 ± 47.2b	60.23 ± 35.64bc	7.50 ± 0.54a	0.27 ± 0.19b	1.5 ± .14ab	23.18 ± 1.78a
	BH-BS2	7.8 ± 0.07a	-278.2 ± 48.1c	-171.7 ± 50.9b	131.14 ± 8.45a	4.45 ± 3.57a	0.31 ± 0.28b	1.61 ± 0.204a	23.33 ± 1.62a
58 days (tillering stage)	TY-S0	6.5 ± 0.34B	510.9 ± 33.8A	438.3 ± 88.5A	61.90 ± 1.10B	26.84 ± 2.63A	4.38 ± 1.87A	2.02 ± 0.05B	9.66 ± 0.05B
	TY-S1	6.5 ± 0.13B	439.8 ± 127.7A	494.3 ± 41.6A	63.57 ± 5.55B	25.12 ± 0.35A	2.03 ± 0.42AB	2.04 ± 0.06A	10.25 ± 0.24B
	TY-S2	6.5 ± 0.12B	536.8 ± 10.9A	507.3 ± 38.6A	69.00 ± 10.23AB	26.34 ± 2.63A	1.85 ± 0.73AB	2.01 ± 0.12B	9.96 ± 0.11B
	TY-BS1	7.2 ± 0.41A	435 ± 67A	501.5 ± 215.7A	81.84 ± 2.01A	30.37 ± 4.73A	1.30 ± 1.81B	2.19 ± 0.09A	13.8 ± 0.94A
	TY-BS2	7.0 ± 0.01A	488.7 ± 33.9A	452.7 ± 203.3A	79.26 ± 11.7A	30.06 ± 4.61A	0.23 ± 0.13B	2.18 ± 0.08A	14.57 ± 1.39A
	BH-S0	8.4 ± 0.12b	434.7 ± 61.5a	423 ± 176.7a	47.06 ± 15.71a	33.12 ± 3.86a	5.23 ± 1.77c	1.12 ± 0.05c	19.42 ± 0.56b
	BH-S1	8.4 ± 0.1b	358.3 ± 117.3ab	334.7 ± 181.7a	34.73 ± 3.84a	33.06 ± 5.14a	7.03 ± 0.37bc	1.41 ± 0.12ab	17.98 ± 0.4b
	BH-S2	8.6 ± 0.07a	205.6 ± 174.6b	279.3 ± 140.3a	39.91 ± 2.90a	29.06 ± 4.07a	11.74 ± 2.05a	1.38 ± 0.17b	18.17 ± 0.19b
	BH-BS1	8.7 ± 0.04a	336.6 ± 24.2ab	563 ± 11a	32.91 ± 5.42a	26.83 ± 2.09a	6.52 ± 3.02bc	1.42 ± 0.14ab	23.17 ± 1.39a
	BH-BS2	8.6 ± 0.03a	206.6 ± 132.9b	229.7 ± 105.6a	39.41 ± 5.57a	29.53 ± 2.42a	9.36 ± 1.21ab	1.62 ± 0.08a	22.37 ± 1.33a
120 days (heading stage)	TY-S0	7.1 ± 0.14C	435.3 ± 24.6A	318 ± 199.3B	124.11 ± 7.62B	43.10 ± 2.20A	1.36 ± 2.06A	2.04 ± 0.04BC	9.85 ± 0.16B
	TY-S1	7.2 ± 0.34BC	380.6 ± 69.1A	161 ± 36.7AB	137.37 ± 25.1AB	41.05 ± 2.73A	0.93 ± 0.51A	2.15 ± 0.10AB	9.58 ± 0.13B
	TY-S2	7.2 ± 0.12BC	352.9 ± 74.8A	227.7 ± 17.9B	148.29 ± 19.82A	43.61 ± 1.30A	0.63 ± 0.11A	2.01 ± 0.03C	9.69 ± 0.25B
	TY-BS1	7.5 ± 0.04AB	446.3 ± 22.9A	385.7 ± 112.3A	174.01 ± 27.15A	41.64 ± 18.14A	0.16 ± 0.17A	2.05 ± 0.11BC	13.34 ± 1.19A
	TY-BS2	7.6 ± 0.19A	437.8 ± 76.9A	465.7 ± 206.4B	144.46 ± 11.16A	31.38 ± 2.56A	0.05 ± 0.04A	2.26 ± 0.02A	13.79 ± 0.46A
	BH-S0	8.3 ± 0.15bc	164.9 ± 7.9b	-10.3 ± 112b	102.80 ± 13.49a	32.09 ± 3.24b	1.57 ± 1.54a	1.1 ± 0.06b	19.81 ± 0.35b
	BH-S1	8.5 ± 0.07a	234.7 ± 93.6ab	129 ± 30.8ab	75.80 ± 10.85ab	29.59 ± 2.63b	0.52 ± 0.06a	1.45 ± 0.109a	18.46 ± 0.61b
	BH-S2	8.4 ± 0.14abc	164.8 ± 60.8b	186.3 ± 78.3a	68.05 ± 12.12b	37.34 ± 2.79a	0.26 ± 0.23a	1.25 ± 0.15ab	18.93 ± 0.29b
	BH-BS1	8.5 ± 0.12ab	321.1 ± 50.3a	210.3 ± 58.9a	80.33 ± 7.14ab	40.25 ± 3.19a	0.33 ± 0.06a	1.49 ± 0.12a	22.52 ± 1.85a
	BH-BS2	8.2 ± 0.05c	251.9 ± 75.1ab	118 ± 112.2ab	100.94 ± 26.30a	41.55 ± 2.31a	1.97 ± 2.17a	1.5 ± 0.16a	21.81 ± 1.38a

*^a^Treatment: TY represents acidic Utisol from Taoyuan, BH represents alkaline Inceptisol from Binhai; control without straw and biochar (S0), with straw at low (S1) and high (S2) rate, and their counterpart plus biochar (BS1 and BS2). ^b^Mean values ± SD, n = 3. The captical letters indicate significant differences in parameters among treatments for TY soils at the individual stage, while the lowercase letters are used for BH soils, analyzed by Duncan’s multiple range test (P < 0.05). “–” means the concentration below detecting limit.*

**Table 2:** Repeat measures ANOVA of rice growing stages and treatments on soil properties and gases emissions.

Soil	Items	Stage (rice growing)	Treatment	Stage × Treatment
TY				
	DOC	*P* < 0.001 ^a∗∗∗^	*P* = 0.005 ^∗∗^	*P* = 0.264 ns
	pH	*P* < 0.001 ^∗∗∗^	*P* < 0.001 ^∗∗∗^	*P* = 0.278 ns
	*Eh*	*P* < 0.001 ^∗∗∗^	*P* < 0.001 ^∗∗∗^	*P* < 0.001 ^∗∗∗^
	${\text{NH}}_{4}^{+}$	*P* < 0.001 ^∗∗∗^	*P* = 0.556 ns	*P* = 0.727 ns
	${\text{NO}}_{3}^{-}$	*P* = 0.034 ^∗^	*P* = 0.027 ^∗^	*P* = 0.163 ns
	TC	*P* = 0.409 ns	*P* < 0.001 ^∗∗∗^	*P* = 0.216 ns
	TN	*P* < 0.001 ^∗∗∗^	*P* = 0.018 ^∗^	*P* = 0.028 ^∗^
	C/N ratio	*P* = 0.184 ns	*P* < 0.001 ^∗∗∗^	*P* = 0.264 ns
	Cumulative CH_4_ emission	*P* < 0.001 ^∗∗∗^	*P* < 0.001 ^∗∗∗^	*P* < 0.001 ^∗∗∗^
	Cumulative N_2_O emission	*P* < 0.001 ^∗∗∗^	*P* < 0.001 ^∗∗∗^	*P* = 0.331 ns
	CH_4_ flux	*P* = 0.022 ^∗^	*P* = 0.026 ^∗^	*P* = 0.043 ^∗^
	N_2_O flux	*P* = 0.011 ^∗^	*P* = 0.240 ns	*P* = 0.509 ns
BH				
	DOC	*P* < 0.001 ^∗∗∗^	*P* = 0.037 ^∗^	*P* < 0.001 ^∗∗∗^
	pH	*P* < 0.001 ^∗∗∗^	*P* = 0.213 ns	*P* = 0.004 ^∗∗^
	*Eh*	*P* < 0.001 ^∗∗∗^	*P* < 0.001 ^∗∗∗^	*P* < 0.001 ^∗∗∗^
	${\text{NH}}_{4}^{+}$	*P* < 0.001 ^∗∗∗^	*P* = 0.414 *ns*	*P* = 0.801 ns
	${\text{NO}}_{3}^{-}$	*P* < 0.001 ^∗∗∗^	*P* = 0.014 ^∗^	*P* < 0.001 ^∗∗∗^
	TC	*P* = 0.255 ns	*P* = 0.001 ^∗∗∗^	*P* = 0.354 ns
	TN	*P* = 0.175 ns	*P* = 0.013 ^∗^	*P* = 0.074 ns
	C/N ratio	*P* = 0.543 ns	*P* < 0.001 ^∗∗∗^	*P* = 0.470 ns
	Cumulative CH_4_ emission	*P* < 0.001 ^∗∗∗^	*P* < 0.001 ^∗∗∗^	*P* < 0.001 ^∗∗∗^
	Cumulative N_2_O emission	*P* < 0.001 ^∗∗∗^	*P* = 0.249 *ns*	*P* = 0.005 ^∗∗^
	CH_4_ flux	*P* < 0.001 ^∗∗∗^	*P* < 0.001 ^∗∗∗^	*P* < 0.001 ^∗∗∗^
	N_2_O flux	*P* = 0.008 ^∗∗^	*P* = 0.593 *ns*	*P* = 0.424 ns

*^a∗∗∗^For the effect, ^∗^, ^∗∗^, ^∗∗∗^ denote significant difference at P < 0.05, P < 0.01, and P < 0.001, respectively. The ns means no significant difference.*

**FIGURE 1:**
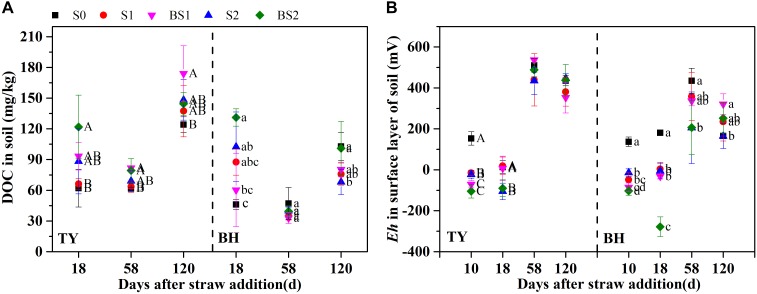
Soil dissolved organic carbon contents **(A)** and redox potential in surface layer of paddy soil **(B)** among the treatments during rice growing season in 2016. Error bars present standard deviation of means (*n* = 3). The different letters (capital letter for the TY soil and lowercase for the BH soil) indicate significant difference among treatments at each stage, which is analyzed by Duncan’s multiple range test (*P* < 0.05).

Similar trends of soil DOC variation were found in both soils (Figure 1A). Soil DOC was greatly increased after straw addition during the seedling stage, while it decreased at the tillering stage. Soil DOC was also increased by the biochar amendment over all rice growth stages, when compared with that in control, except for a decrease at the tillering and heading stages in the BH soil (Figure 1A), which might have been caused by adsorption on the biochar. The dynamics of soil redox potential (*Eh*) was generally consistent in the two paddy soils (Figure 1B). The *Eh* was generally low during the flooding period, and sharply increased through the drainage. Straw incorporation reduced the *Eh* in both soils during the flooding stage, which ranged from −104.7 to −15.2 mV in the TY soil, and from −103.6 to −13.7 mV in the BH soil. A lower *Eh* in both soils was recorded at the seedling stage with biochar amendment, which was nearly 36–272 mV lower than treatments without biochar incorporation (S1 and S2) (Figure 1B). Moreover, the difference in *Eh* between the treatments with and without biochar amendment became smaller following drainage.

### CH_4_ Emissions From Rice Paddy Soils

**FIGURE 2:**
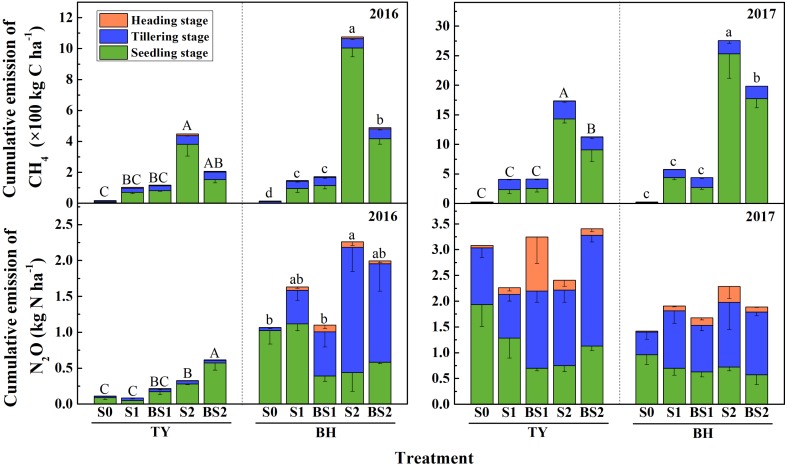
Cumulative emissions of CH_4_ and N_2_O over the rice growing season in 2016 and 2017. Colors in column denote different stage: the seedling stage in green, the tillering stage in blue and the heading stage in orange. Error bars present standard deviation of means (*n* = 3). The different letters (capital letter for the TY soil and lowercase for the BH soil) among different treatments indicate significant difference of the cumulative emissions during the whole rice growing season, which was analyzed by Duncan’s multiple range test (*P* < 0.05).

The methane fluxes showed significant differences among treatments and varied over the rice growing season (Supplementary Figure 1). In general, the transient and cumulative CH_4_ emissions in the TY soil were much lower than those in the BH soil. During the rice growing season in 2016, CH_4_ emissions were more concentrated in the seedling stage in both soils (Figure 2 and Supplementary Figure 1), which accounted for 64.5–93.4% of cumulative methane emissions in all treatments except the control (S0) (Figure 2).

CH_4_ emissions significantly increased in both soils with straw addition (*P* < 0.01), and the response in the high straw rate (S2) was greater than in the low straw rate (S1) (Figure 2 and Supplementary Figure 1). The S2 treatment had the highest cumulative CH_4_ emissions among all the treatments with 448 kg C ha^−1^ in TY and 1,075 kg C ha^−1^ in BH in 2016. In contrast, the cumulative CH_4_ emissions significantly decreased to 207 kg C ha^−1^ in the TY soil and 489 kg C ha^−1^ in the BH soil under biochar amendment at the high straw input level (BS2) (*P* < 0.05). However, no significant difference in cumulative CH_4_ emissions was detected between with and without biochar amendment at the low straw input level, i.e., S1 and BS1 (Figure 2). Furthermore, the data of CH_4_ emissions collected in the rice growing season in 2017 were highly consistent with that in 2016, showing a significant increase by rice straw addition and a significant suppression by biochar amendment in the high straw incorporated soils (Figure 2 and Supplementary Figure 1, *P* < 0.05).

### N_2_O Emissions From Rice Paddy Soils

A similar trend of N_2_O flux among all the treatments was observed in the two soils during the rice growing season. For both soils in the 2016 rice growth season, the N_2_O flux was pronounced at the start of the continuously flooding period, and quickly decreased within a week (Supplementary Figure 2a). No marked variation was found in the following drainage and re-flooding periods, except for a peak flux in the BH soil at the 55th day, which might be due to the alternation of the water regime caused by a rainfall event.

Generally, the N_2_O flux in the TY soil was slightly lower than that in the BH soil in 2016 (Supplementary Figure 2a). During the whole rice growing season in 2016, cumulative N_2_O emissions in the TY soil were significantly lower than those in the BH soil (Figure 2). For the TY soil, nearly 55.4–92.8% of the cumulative N_2_O was emitted at the seedling stage. Moreover, the cumulative N_2_O emissions significantly increased with straw addition in treatment S2 by 1.94-fold (*P* < 0.05), while little effect was seen in treatment S1. The cumulative N_2_O emissions increased by 0.88- to 1.51-fold with biochar addition, compared with no biochar incorporation (Figure 2). For the BH soil, 91.2–99.8% of the cumulative N_2_O emissions originated at the seedling and tillering stages. The cumulative N_2_O emissions in S2 treatment was significantly higher than that in control (S0, *P* < 0.05), while a decreasing trend was observed with biochar amendment in treatment BS2 (Figure 2). For both TY and BH soil, N_2_O emissions showed no significant difference among treatments in the year 2017 (Supplementary Figure 2b).

### Abundances of Methanogens and Methanotrophs in Rice Paddy Soils

**FIGURE 3:**
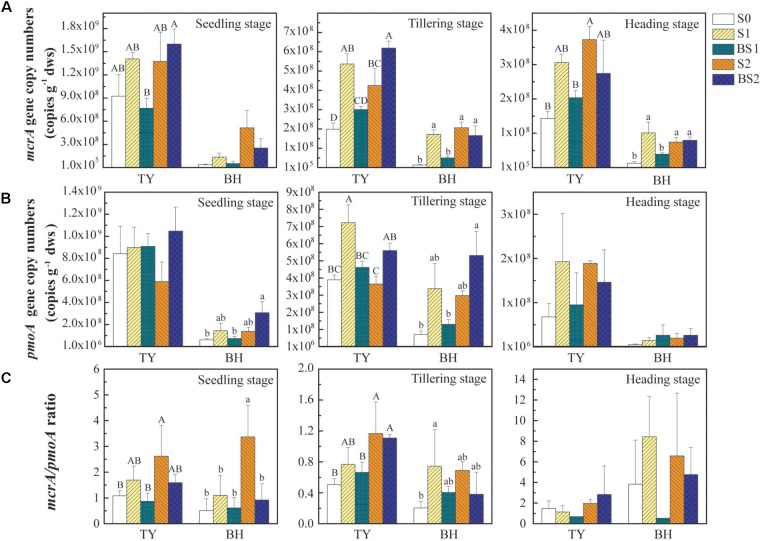
The abundance of *mcrA* **(A)** and *pmoA* **(B)** genes and the *mcrA*/*pmoA* ratio **(C)** at the seedling, tillering, and heading stages in 2016. Error bars present standard deviations of means (*n* = 3). The different letters (capital letter for the TY soil and lowercase for the BH soil) indicate significant difference among different treatments at each sampling point, which was analyzed by Duncan’s multiple range test (*P* < 0.05).

The abundances of *mcrA* and *pmoA* genes, encoding the key enzymes functioning in the generation of CH_4_ and the consumption of CH_4_, respectively, were quantified to estimate the dynamics of methanogens and methanotrophs during the rice growing season. Generally, both *mcrA* and *pmoA* genes were more abundant in the TY soil (ranged from 1.43 × 10^8^ to 1.60 × 10^9^ copy genes g^−1^ dws for *mcrA*, and from 6.79 × 10^7^ to 1.05 × 10^9^ copy genes g^−1^ dws for *pmoA*) than those in the BH soil (ranged from 1.27 × 10^7^ to 5.15 × 10^8^ copy genes g^−1^ dws for *mcrA* and from 5.05 × 10^6^ to 3.38 × 10^8^ copy genes g^−1^ dws for *pmoA*), and decreased over time in both soils (Figures 3A,B). The *mcrA* gene abundance generally increased with straw addition alone (S1 and S2) and high straw level plus biochar (BS2), and showed statistically significant differences at tillering and heading stages for both soil types (Figure 3A, *P* < 0.05). Interestingly, the *mcrA* gene abundance in biochar amendment under low straw input (BS1) showed no significant difference with control (S0), but was generally lower than S1 treatment (Figure 3A, *P* < 0.05), which could be due to the suppressive influence of the biochar amendment under low straw input.

Compared with the control (S0), straw addition at low and high rates (S1 and S2) showed no significant promotion or suppression effects on the *pmoA* gene abundances in both TY and BH soils, except that *pmoA* gene abundance was significantly higher in S1 than in S0 at tillering in the TY soil (Figure 3B). By contrast, biochar amendment under high straw input (BS2) showed a visible promotion on the *pmoA* gene abundance in both TY and BH soils at seedling and tillering stage, when compared with S0 and S2 treatments. A distinct augment of *pmoA* gene abundance by 53.3–123.9% was observed in the BS2 treatment at the seedling and tillering stages, when compared with S2 in both soils (Figure 3B). Consequently, biochar plus straw amendment (BS1 and BS2) generally decreased the ratio of *mcrA* to *pmoA* gene abundance in comparison with straw addition alone (S1 and S2) (Figure 3C).

Correlation analysis showed that *mcrA* gene abundance was positively correlated with the CH_4_ flux (*r* = 0.514, *P* < 0.01 for the TY soil, *r* = 0.730, *P* < 0.01 for the BH soil), while negatively correlated with soil pH and *Eh* in both soils (Supplementary Table 2). The *pmoA* gene abundance showed no significant correlation with the CH_4_ flux in both soils, but was negatively correlated with *Eh* (*r* = −0.665, *P* < 0.01) was observed in the TY soil, but not in the BH soil (Supplementary Table 2). Otherwise, the ratio of *mcrA* to *pmoA* gene abundance was positively correlated with the cumulative CH_4_ emission (*r* = 0.476, *P* < 0.01 for the TY soil, *r* = 0.299, *P* < 0.05 for the BH soil (Supplementary Table 2). All these suggested that the *mcrA* gene abundance, compared with *pmoA* gene abundance, was more closely related to the dynamics of CH_4_ flux, and the ratio of *mcrA* to *pmoA* gene abundance also could be a good indicator for CH_4_ flux.

### Abundances of N_2_O-Related Functional Genes

**FIGURE 4:**
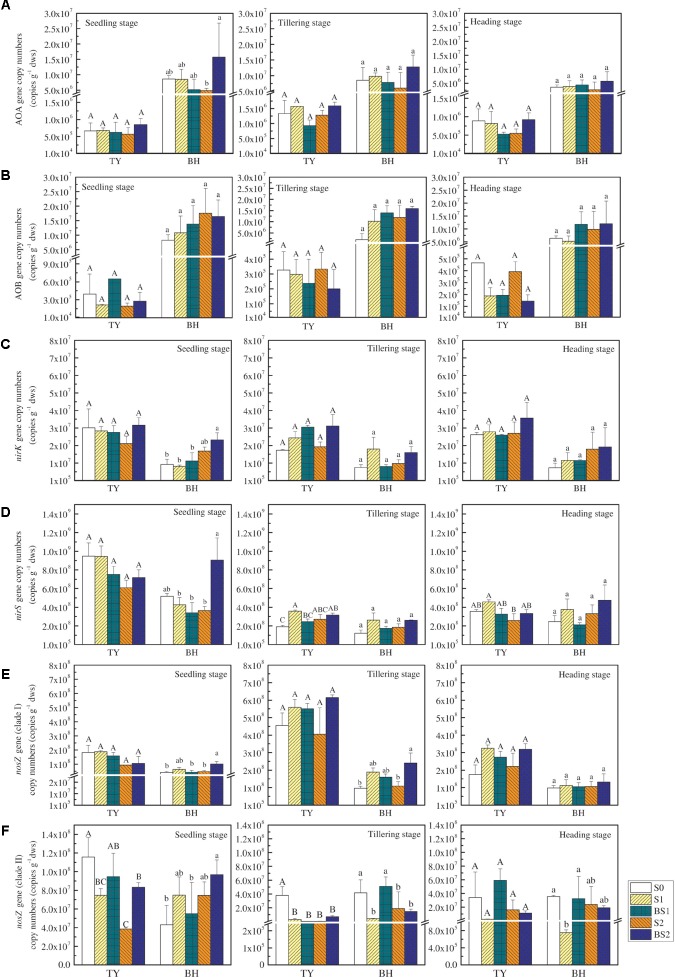
The abundance of N_2_O-related functional genes at the seedling, tillering and heading stages in 2016. The copy numbers of archaeal *amoA*, bacteria *amoA*, *nirK*, *nirS*, *nosZ* I, and *nosZ* II genes are exhibited in **(A–F)**, respectively. Error bars present standard deviations of means (*n* = 3). The different letters (capital letter for the TY soil and lowercase for the BH soil) indicate significant difference among different treatments at each sampling point, which was analyzed by Duncan’s multiple range test (*P* < 0.05).

The functional genes relevant to the N_2_O production and consumption were analyzed in this study (Figure 4). The abundance of ammonia-oxidizing archaea (AOA) and bacteria (AOB) *amoA* genes were both lower in the TY soil (ranged from 5.29 × 10^5^ to 1.59 × 10^6^ copy genes g^−1^ dws for AOA and from 1.44 × 10^5^ to 6.48 × 10^5^ copy genes g^−1^ dws for AOB) compared with that in the BH soil (ranged from 2.65 × 10^6^ to 1.57 × 10^7^ copy genes g^−1^ dws for AOA and from 2.03 × 10^6^ to 1.75 × 10^7^ copy genes g^−1^ dws for AOB), with slight change over the crop growth stages (Figures 4A,B). Besides, no significant variations of AOA and AOB *amoA* gene abundances were observed among all the treatments regardless of rice growth stage in both soils, except for a significant promotion of AOA abundance in the BS2 treatment at the seedling stage in the BH soil (Figures 4A,B). Generally, both straw addition and biochar incorporation (S1, S2, BS1, and BS2) had little impact on AOA and AOB *amoA* gene abundances for both soil types over time, when compared with the control (S0).

Similarly, straw addition and biochar application showed little effect on the *nirK* gene abundance, except for a significant increase by 1.51-fold in BS2 treatments in the BH soil at the seedling stage (*P* < 0.05), when compared with the control (S0) (Figure 4C). In general, the abundance of the *nirS* gene (ranged from 1.22 × 10^8^ to 9.48 × 10^8^ copy genes g^−1^ dws) was much greater than that of *nirK* gene (ranged from 7.28 × 10^6^ to 3.28 × 10^7^ copy genes g^−1^ dws) (*P* < 0.01). Straw addition alone (S1, S2) or biochar amendment (BS1, BS2) had little effect on the *nirS* gene abundance compared with the control (S0) for both soils over time, except for significant increases by 87.6% and 65.8% under the treatments S1 and BS2 in the TY soil at the tillering stage, respectively (Figure 4D, *P* < 0.05).

The *nosZ* gene, as an index of the nitrous oxide-reducing bacteria, consisted of two distinct clades (clade I and clade II). The abundance of *nosZ* clade I was higher in both soils at tillering compared to the seedling stage, followed by a decrease at the heading stage (Figure 4E). Straw addition alone (S1, S2) had no significant effect on the abundance of the *nosZ* I gene for both TY and BH soils. Biochar amendment (BS1, BS2) showed no obvious influence on the *nosZ* I gene abundance in the TY soil, while BS2 treatment significantly increased the *nosZ* I gene abundance in the BH soil at the seedling and tillering stages (Figure 4E, *P* < 0.05), during which N_2_O emissions peaked. The enhanced *nosZ* I gene abundance could be responsible for the suppression of N_2_O emissions in BH soil. The abundance of *nosZ* II gene showed slight variation over time, and generally decreased in straw addition treatments (S1 and S2) in relative to S0 in both soils (Figure 4F). Biochar amendment under low straw addition (BS1) significantly buffered the straw-induced decrease of *nosZ* II gene in the BH soil at tillering and heading stages, but showed no significant effect in the TY soil, which further explained the suppression of N_2_O emissions in BH soil.

### Community Similarity of the Methanotroph and N_2_O-Reducing Bacteria

The methanotroph community at seedling stage and *nosZ* gene containers community at seedling, tillering and heading stages were characterized by Miseq sequencing. After resampling, 5,689 *pmoA* gene reads and 8,356 *nosZ* gene reads from each sample were selected for alpha- and beta-diversity analysis. Alpha diversity of both methanotroph and *nosZ*-containing bacteria showed no significant differences among treatments in both soils. However, the alpha diversity of *nosZ*-containing bacteria was generally much higher in the BH soil than in the TY soil (Supplementary Table 3, *P* < 0.05).

**FIGURE 5:**
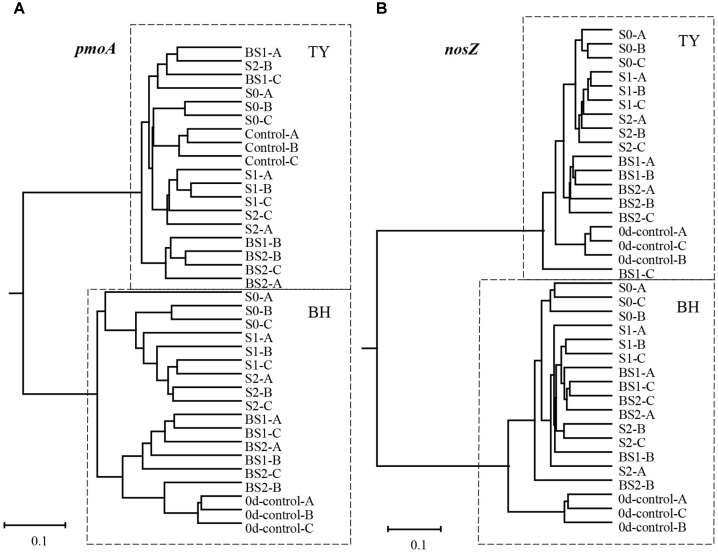
UPGMA dendrogram contrasted from Bray–Curtis distance matrix of *pmoA* **(A)** and *nosZ* I **(B)** gene sequences at day 18 during the seedling stage. 0d-control represents the background of original soil at the beginning of pot experiment, and A, B, C mean three replicate pots.

The UPGMA clustering analysis for the beta-diversity of methanotroph and *nosZ* gene container communities at the seedling stage showed a clear separation between the two soil types (Figure 5). Within each soil type, samples generally clustered among treatments, with clear separation between treatments with and without biochar amendment for methanotroph community in the BH soil, and for *nosZ* gene container in the TY soil (Figure 5). These results suggested that community structure of methanotroph and *nosZ* gene containing denitrifiers were largely influenced by the addition of biochar than that of the straw, depending on the soil type.

**FIGURE 6:**
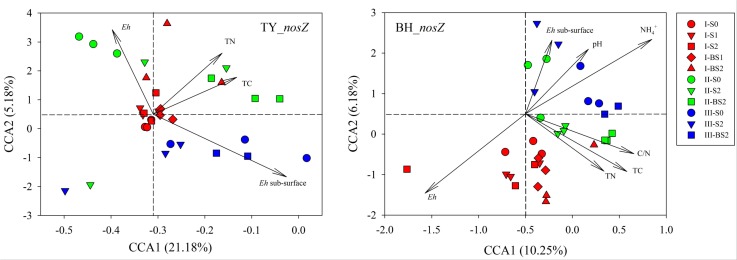
Canonical correlation analysis for the *nosZ* gene containing denitrifier communities and soil properties. Three rice growth stages, seedling, tillering and heading, were included and labeled as I (red), II (green) and III (blue), respectively.

Canonical correlation analysis based on the OTU matrix was performed to examine the influence of soil environmental factors on the community composition of *nosZ* gene containing denitrifiers. On the CCA plots, well separation of *nosZ* gene community among the three rice growth stages but slight aggregation among treatments were observed (Figure 6). The *x*-axis explained 21.18% and 10.25% of the variation of *nosZ* gene community in TY and BH soils, and the *y*-axis explained 5.18% and 6.18% of variation, respectively. Monte Carlo tests showed that *Eh*, TN and TC were factors significantly influencing *nosZ* gene containing community in the TY soil, and together explained the variation by 11.85%. For the BH soil, *Eh*, TC, TN, C/N, pH and ${\text{NH}}_{4}^{+}$ significantly influenced *nosZ* gene containing community and together explained 24.05% of variation (Figure 6). For both soils, *Eh* generally accounted for the greatest impact on *nosZ* gene containing community (Supplementary Table 4).

### Community Composition of Methanotroph and N_2_O-Reducing Bacteria Under Different Treatments

**FIGURE 7:**
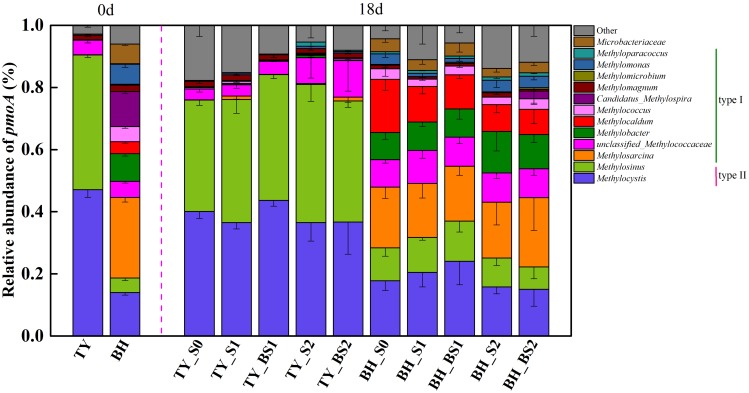
Taxonomic distribution of *pmoA*-based methanotrophs at the genus level at day 18 during the seedling stage. Other includes the sequences with a relative abundance less than 0.9% and the unclassified sequences at genus level. Mean ± SD, *n* = 3. 0d represents the original soil used in this experiment.

Analysis of methanotroph communities based on *pmoA* gene further showed that the TY soil and the BH soil possessed different methanotroph communities, with the TY soil dominated by Type II methanotrophs and the BH soil by type I methanotrophs (Figure 7). For the TY soil, *Methylocystis* (36.53–47.09%) and *Methylosinus* (38.93–44.46%) of Alphaproteobacteria (type II) were the dominant group and showed no marked variations among the five treatments, while the proportion of unclassified_*Methylococcaceae* belonging to type I increased from 3.54% in control (S0) to 8.27% in S2 and 11.78% in BS2 treatment, respectively. For BH soil, type II methanotrophs (*Methylocystis* and *Methylosinus*) accounted for 25.08–37.01% of the methanotroph community and type I methanotrophs accounted for 53.13–63.55%, with 3.93–13.67% of unclassified among the five treatments in day 18 (Figure 7). Compared with the S0, S1, and BS1 treatments, the proportion of type II methanotrophs decreased by 14.7–40.12% while the proportion of *Methylobacter* (type I methanotroph) significantly increased by 21.30–53.13% with the high straw incorporation (S2 and BS2). Straw addition and biochar amendment (S1, S2, BS1, and BS2) both decreased the relative abundance of *Methylocaldum* by 33.26–52.61% compared with the control (S0). Besides, a distinct increased (by 14.03%) of *Methylosarcina* was observed in the BS2 treatment with biochar amendment at high straw input. All these suggested that methanotrophs community responded to straw and biochar amendments more greatly in the BH soil than in the TY soil, coinciding with the separation of methanotrophs among the treatments in the UPGMA dendrogram.

**FIGURE 8:**
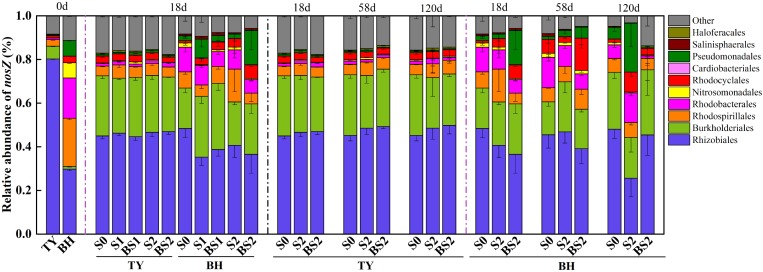
Taxonomic distribution of *nosZ* I gene derived OTUs at the order level in paddy soils over time. Other includes the sequences with a relative abundance less than 0.8% and the unclassified sequences. Mean ± SD, *n* = 3. 0d represents the original soil used in this experiment.

As for typical *nosZ* genes, 1,362 OTUs at 97% similarity level were identified from 72 samples covering all treatments at seedling stage, and S0, S2, and BS2 treatments over three rice growth stages. The majority of *nosZ* gene reads were grouped into *Proteobacteria* (89.7%), with 3.48–18.05% unclassified (Figure 8). At the order level, the *nosZ* gene community was predominated by *Rhizobiales* in the TY soil with a proportion of 80.27%, while it was dominated by *Rhizobiales*, *Rhodospirillales*, and *Rhodobacterales* with similar proportions between 18.47% and 29.76% in the BH soil at day 0 (Figure 8). Flooding changed the *nosZ* gene community with *Rhizobiales* significantly decreasing from 80.27 to 44.61–46.98% in the TY soil, *Rhodospirillales* decreasing from 22.09 to 4.87–15.04% in the BH soil, and *Burkholderiales* increasing from 1.20–5.85% to 18.55–30.32% in both soils. After flooding, the *nosZ* gene containing denitrifiers showed no significant variation among all the treatments over the time in the TY soil (Figure 8). In contrast, the *nosZ* gene containing denitrifiers community in the BH soil showed visible variations among different straw and biochar treatments and greater variation over the three rice growth stages. Within treatments at day 18 (seedling stage), the proportion of *Burkholderiales* significantly increased by 6.78–50.28% in the straw addition alone treatment (S1 and S2), and 24.53–63.43% in the biochar amendment treatments (BS1 and BS2) compared with the control (S0) (Figure 8) in the BH soil. Compared with the S0 treatment, S2 treatment had a higher proportion of *Pseudomonadales* and *Rhodobacterales* but a relatively low proportion of *Rhizobiales* at day 120 (heading stage), while the proportions of these groups in BS2 treatment were closer to that in S0 treatment (Figure 8).

## Discussion

### Effect of Straw Addition and Biochar Incorporation on CH_4_ Emissions

In this study, the transient CH_4_ and N_2_O fluxes showed quite similar patterns between the two different soil types. Both the CH_4_ flux and cumulative emissions peaked at the early rice growth period (seedling stage), and a significant increase in cumulative CH_4_ emissions induced by the rice straw amendment and a greater increase with high straw rate input (S2 treatment) were observed (Figure 2). Correspondingly, a significantly higher DOC in the S2 treatment than in the S0 and S1 treatments (no straw and low straw rate) were detected in the early rice growth period (Table 1), indicating that more straw-driven C was probably transforming into CH_4_ at the seedling stage. As suggested by previous studies, the increase of CH_4_ emissions could be attributed to the additional substrate (e.g., H_2_/CO_2_ and acetate) provided for the methanogens via anaerobic decomposition of crop residues (Watanabe et al., 1995, 1998; Ma et al., 2007; Wang et al., 2016). The amounts of straw used in this study were equal (S1) and double (S2) to all aboveground biomass return, and the high straw level (S2) was set to stimulate the straw concentrated patches in field, as straw is generally surface returned to the field or incorporated into the plow layer by plowing thus cause highly concentrated patches. The similar case that the higher straw amount induced higher CH_4_ and N_2_O emissions observed in our pot experiments probably occurred in the field, so the amount of straw return to field should be taken into consideration in practice, even though biochar amendment could markedly mitigate the CH_4_ emissions in straw incorporated soils.

On the other hand, soil *Eh* was extremely low in the BH soil (−104 to −14 mV in surface and −190 to −156 mV in subsurface) and the TY soil (−105 to −15 mV in surface and −165 to −146 mV in subsurface) 1 week after straw input (Figure 1B and Supplementary Figure 3), and significantly negative correlations were observed between soil *Eh* and CH_4_ fluxes in both soils (Supplementary Table 2). This is consistent with previous studies which showed that soil *Eh* ranging from −230 to −150 mV greatly favored CH_4_ emissions (Wang et al., 1993). Moreover, the soil *Eh* decrease was considered as another main reason of the enhanced CH_4_ emissions after rice straw amendment, as more electron donors were provided for methanogen process under low *Eh* conditions (Tanji et al., 2003; Ma et al., 2008; Shen et al., 2014). Soil *Eh* therefore could be a sensitive indicator for CH_4_ emission forecasting under flooded conditions.

By contrast, biochar amendment significantly decreased the CH_4_ emissions under the high straw rate during the whole rice growing season in this study. Though sporadic studies found that biochar amendment increased CH_4_ emissions in paddy fields (Knoblauch et al., 2011; Zhang et al., 2012), the majority of previous studies have shown that single biochar application could decrease CH_4_ emissions effectively, and this was attributed to the increased soil pH induced by biochar (Tanji et al., 2003; Conrad and Klose, 2006; Liu et al., 2011; Shen et al., 2014; Ly et al., 2015; Thammasom et al., 2016). In this study, a significant pH increase by 0.5 unit with biochar amendment (BS2) was also observed in the acidic TY soil at the early period (at day 18 and day 58), which partially accounted for the decreased CH_4_ emissions under biochar amendment with high straw input (Table 1). However, biochar amendment did not significantly increase pH in the BH soil at both straw input levels (Table 1), probably due to the alkaline property of the BH soil. Biochar-induced pH increase therefore could not explain the decreased CH_4_ emissions in the BS2 treatment in the BH soil. Moreover, though biochar amendment with straw incorporation markedly decreased soil *Eh* for both soil types, while CH_4_ emissions did not increase with decreasing *Eh* as observed in the straw incorporation treatments (S1 and S2). Biochar contains electroactive functional groups such as quinone/hydroquinone and has been shown to serve as electron acceptors or donors during the redox processes (Kluepfel et al., 2014). It is also proposed as a “geoconductor” which could directly transfer electrons from char matrices to minerals (Sun et al., 2017). The depressed CH_4_ emissions from biochar amended soil under high straw incorporation in this study therefore could be explained as a result of biochar competing for electrons with CO_2_ thus disturbing the methanogenesis process. This also explained why the depression of biochar on CH_4_ emissions was only obvious under high straw but not under low straw inputs, as the high straw input created a stronger redox condition (much lower *Eh* in BS2 than BS1, Table 1) and biochar could trap more electrons. All these suggest that biochar amendment together with straw incorporation is beneficial to mitigating CH_4_ emissions from paddy soils, especially under high straw input conditions.

On the other hand, CH_4_ emissions from soil are dependent on the balance of microbe-mediated methanogenesis and methane oxidation processes. Methane produced via methanogenesis under anaerobic conditions could be consumed by the methanotrophic bacteria via oxidizing CH_4_ to CO_2_, when O_2_ was available (Bridgham et al., 2013). Some previous studies showed that straw incorporation enhanced CH_4_ emissions with an increase in the abundance of the *mcrA* gene (Freitag et al., 2010; Cai et al., 2017). Consistently, straw addition generally increased the abundance of the *mcrA* gene, but showed no significant effect on the abundance of the *pmoA* gene in this study (Figures 3A,B). As a consequence, the *mcrA*/*pmoA* gene abundance ratio increased with straw incorporation, and both the ratio and the *mcrA* gene abundance were positively correlated with CH_4_ emissions in both soils (Supplementary Table 2), which could be attributed to the stimulation of straw degradation and high available DOC for methanogens (Figure 2 and Table 1). Biochar amendments under high straw input (BS2) showed no clear effect on the *mcrA* gene abundance but promoted the *pmoA* gene abundance, and consequently decreased the ratio of *mcrA*/*pmoA* significantly in both soils, when compared with that of S2 (Figure 3C). Therefore, it was the activated methanotrophs and the attenuated ratio of *mcrA*/*pmoA* that lead to the suppressed CH_4_ emissions after biochar amendment at high straw incorporation.

### Effect of Straw Addition and Biochar Incorporation on N_2_O Emissions

For both TY and BH soils, straw amendment at the low rate (S1) caused no significant increase in N_2_O emissions while the cumulative N_2_O emissions increased significantly with straw addition at the high rate (S2) in this study (Figure 2). Ambiguous effects of straw amendment on N_2_O emissions in paddy soils had been found in previous studies. For example, Ma et al. (2007) found that the N_2_O emissions decreased by approximately 30% with straw incorporation, while significant N_2_O emissions increased with straw addition was observed in other studies (Ma et al., 2009; Hu et al., 2016). The different effects of straw application on N_2_O emissions were mainly due to the quality of the crop residues with various C/N ratios (Toma and Hatano, 2007). Incorporating crop residue with a high C/N ratio (>40) (like wheat straw) could enhance microbial N immobilization, which results in less available N for nitrification and denitrification (Vigil and Kissel, 1991; Millar and Baggs, 2005; Toma and Hatano, 2007; Rizhiya et al., 2011). In contrast, a lower C/N ratio of straw (like soybean stem, cabbage, and red clover) would provide more available N for denitrifiers and thus result in increased N_2_O emissions (Baruah et al., 2016). Moreover, a negative correlation was detected between the N_2_O emissions from crop residue incorporated soil and straw C/N ratio (Millar and Baggs, 2005), suggesting that the C/N ratio of incorporated straw might be a key factor influencing the N-cycling in paddy soils. In this study, no visible increase in N_2_O emissions was observed under low straw input rate, and significantly higher N_2_O emissions under high straw input rate were only observed in 2016, but not in 2017. The high C/N ratio at about 38 in the rice straw used in this study well explained the non-significant increase in N_2_O emissions under low and high straw input in most cases. The higher N_2_O emissions in S2 than in S0 treatment in 2016 could be attributed to the additional C and N substrate via straw decomposition under high straw input rate, while the effect of straw on N_2_O emissions would be limited in the tested soils.

The cumulative N_2_O emissions in biochar amendment treatments (BS1 and BS2) showed a decreasing trend in the alkaline BH soil. Conversely, N_2_O emissions in BS1 and BS2 in the TY soil showed an increasing trend for two growth seasons and were statistically significant higher compared with straw incorporation treatments (S1 and S2) in 2016. Similarly, some previous studies reported that soil N_2_O emissions decreased significantly following biochar amendment (Liu et al., 2012; Zheng et al., 2012; Saarnio et al., 2013), while some others showed a significant increase of N_2_O emissions after biochar inputs (Verhoeven and Six, 2014). The inconsistent effect of biochar amendment on N_2_O emissions might be explained by the soil properties (Cayuela et al., 2014). The above mentioned studies attributed the reduced N_2_O emissions in the paddy fields with biochar amendment to soil aeration improvement after biochar application and the decrease of ${\text{NH}}_{4}^{+}$ availability due to the absorption by biochar (Lehmann et al., 2006; Zhang et al., 2010). These reasons could well explain the decreasing trend of N_2_O emissions in biochar treatment in the BH soil in this study, but not for the TY soil with a converse trend. Some studies also suggested that the increase of soil pH in biochar-treated soils could enhance the activity of N_2_O reductase within denitrifier microorganisms, and thus reducing the ratio of N_2_O/N_2_ (Yanai et al., 2007). Though soil pH increased significantly by more than 0.5 units in the TY soil under biochar amendments, biochar application did not decrease N_2_O emissions but promoted N_2_O emissions to some extent in this study. The possible explanation for such inconsistency could be: The TY soil contained much higher ammonia concentration (52.91 mg kg^−1^ in BS1 and 45.61 mg kg^−1^ in BS2) than the BH soil (7.50 mg kg^−1^ in BS1 and 4.45 mg kg^−1^ in BS2). The increased soil pH induced by biochar probably stimulated the nitrification and denitrification under such high ammonia condition, thus induced N_2_O emissions in the TY soil. Some studies also suggested that biochar-induced increase of ${\text{NH}}_{4}^{+}$ or ${\text{NO}}_{3}^{-}$-N content was the main reason for the increased N_2_O emissions (Yoo and Kang, 2012; Shen et al., 2014). Differently, our study did not observe significant increase ${\text{NH}}_{4}^{+}$-N content induced by pH improve in the TY soil with biochar amendment, as the high ${\text{NH}}_{4}^{+}$ -N background probably buffered it. These observations suggested that biochar-induced pH increase would not necessarily decrease N_2_O emissions, but might increase N_2_O emissions conversely when available N is high in soil environment. As biochar amendment might produce inconsistent effect on N_2_O emissions in different soils, its extensive application requires appropriate estimation based on soil property.

A previous study found that the increased N_2_O emissions were closely related with the significant increase in AOB abundance after biochar amendment in a paddy soil (Lin et al., 2017). However, in present study, no significant effects of straw and biochar addition on the abundances of AOA and AOB *amoA* genes were found during the rice growth stages (Figure 4), suggesting that nitrification was probably not the main process influencing the N_2_O emissions in both soils. Meanwhile, straw addition showed little effect on the *nirK*, *nirS*, and *nosZ* I genes abundances, but showed depressive effect on *nosZ* II gene abundance in both soils. Previous studies have indicated that the *nosZ* II gene-containing denitrifier had higher affinity to N_2_O than the *nosZ* I gene container and might be more responsible for the mitigation of N_2_O emission (Jones et al., 2014; Yoon et al., 2016). The depression of *nosZ* II gene by straw addition in two soils partially explained the higher N_2_O emissions in S1 and S2 treatment in relative to the control (S0). On the contrary, biochar amendment under high straw input significantly increased the *nirS* and *nosZ* I gene copy numbers, and biochar amendment under low straw input showed promotive effect on *nosZ* II gene in the BH soil (Figure 4). The increased *nosZ* gene abundance probably stimulated the transformation process from N_2_O to N_2_, and thus decreased N_2_O emissions in the BH soil with biochar amendment. As the functional genes were quantified at DNA level and multiple genes were involved in the processes of N_2_O production and consumption, the targeted genes and DNA-based analysis in this study might not sensitively indicate the microbial activity in N-cycling in this study. Further studies at RNA level and based on more functional genes like fungal, archaeal *nirK* and non-typical *nosZ* genes were necessary to reveal the microbial mechanism of N_2_O emissions under straw and biochar amendments in future.

### Effects of Straw and Biochar Addition on Functional Microbial Community

Generally, distinct dominant methanotrophs and *nosZ*-containing denitrifiers groups were found in the TY and BH soil. Particularly, type II methanotrophic groups (i.e., *Methylocystis* and *Methylosinus*) dominated in the TY soil, while the type I methanotrophs (i.e., *Methylosarcina*, *Methylobacter* and *Methylocaldum*) dominated in the BH soils (Figure 7). It has been suggested that both type I and type II were active methanotroph groups in different paddy soils, and that their distributions were mainly determined by the property of original soil types (Kolb et al., 2003; Ho et al., 2011, 2015). Generally, the type I methanotrophs possessed a lower affinity with CH_4_ therefore preferred the condition with lower O_2_ and high CH_4_ concentrations, while the type II methanotrophs were more active in low CH_4_ concentration environments (Dunfield et al., 1999; Macalady et al., 2002). Type I methanotrophs were also interpreted as r-type life strategy which could respond fast to environment change and devote to the oxidation of CH_4_, while the type II were described as K-type life strategy possessing high competition ability under low nutrient conditions (Steenbergh et al., 2010). These characteristics well explained why straw addition resulted in a distinct shift of methanotrophs community in the BH soil but posed little effect in the TY soil in this study, as the BH soil and TY soil were dominated by type I and type II methanotrophs, respectively.

Specifically, the relative abundance of type I *Methylobacter* increased significantly in all straw addition treatments (S1, S2, BS1, and BS2) in the BH soil, corresponding to the significant decrease of nitrate in these treatments (Figure 7 and Table 1). It has been found that the activities of *Methylobacter* can be strongly suppressed by extra ${\text{NH}}_{4}^{+}$ and ${\text{NO}}_{3}^{-}$ supply (King and Schnell, 1994). Markedly decreased ${\text{NO}}_{3}^{-}$ under straw incorporation treatments might relieve the suppression of nitrate and promoted the growth of *Methylobacter*, which well explained the enhanced proportion of *Methylobacter* under straw amendment condition.

On the other hand, biochar amendment in the high straw input treatment (BS2) greatly changed the community composition of methanotrophs, with *Methylosarcina* significantly increased (Figure 7). It has been reported that *Methylosarcina* and *Methylomonas* possibly required a certain O_2_ and relatively higher concentration of CH_4_ for methane oxidation (Lee et al., 2014). DNA-SIP experiment also demonstrated that *Methylosarcina* dominated under high CH_4_ conditions (Zheng et al., 2014). As biochar could adsorb O_2_ or CH_4_, thus creating high-CH_4_ hotspots (Brassard et al., 2016), which might contribute to the increased proportions of *Methylosarcina* in BS2 treatment. Moreover, significantly higher DOC and TN were detected in the BS2 treatment in this study (Table 1), which might contribute to the variation of methanotrophs community. Indirectly, the huge surface area and pores in biochar could provide habitats for microbial activities (Gul et al., 2015). All these difference in soil conditions induced by biochar amendment resulted in the change of methanotrophs community.

Similar to methanotrophs community, the community structure of *nosZ* gene containing bacteria responded to straw and biochar inputs differently in two soil types. For the TY soil, straw addition and biochar amendment showed little effect on the community composition of *nosZ* gene communities (Figure 8). Though straw and biochar additions significantly increased soil DOC, *Eh* in the subsurface of the TY soil was identified as the most significant environmental factor contributing to the shift of community structure in the TY soil in RDA analysis (Figure 6). Similarly, Richardson et al. (2009) found that denitrifiers containing *nosZ* gene were impressible to the dynamics of soil *Eh*. Contrastingly, the *nosZ* gene containing bacteria in the BH soil showed visible variations among different straw and biochar treatments. The relative abundances of *Rhizobiales* and *Nitrosomonadales* were obviously decreased, while *Rhodocyclales* and *Burkholderiales* were increased under straw incorporation in comparison with control. Biochar application (BS1 and BS2) further enhanced the relative abundance of *Burkholderiales*, when compared with the straw input alone treatment (S1 and S2). In a DNA-SIP microcosm experiment, *Burkholderiales* and *Rhodospirllales* were identified as the predominant population under suitable N_2_O reduction conditions, and were responsible for reduction of N_2_O in rice paddy soils (Ishii et al., 2011). Another study also found that denitrifiers belonging to the orders of *Burkholderiales* and *Rhodocyclales* showed strong denitrifying activities in paddy soils (Ishii et al., 2009). The enhanced proportion of *Burkholderiales* and *Rhodocyclales* with biochar amendment might contribute to a more intensive N_2_O consumption, thus led to the decreased N_2_O emissions under biochar amendment in the BH soil.

## Conclusion

2-year pot experiment in this study demonstrated that the rice straw amendment could significantly increase the cumulative CH_4_ emissions in an acidic Utisol (TY) and an alkaline Inceptisol (BH) paddy soil, while biochar amendment could markedly mitigate the CH_4_ emissions augmented by high straw incorporation in both soil types. These results could be explained by the straw-driven C and N substrate change, biochar-induced pH and *Eh* change, or electron competition etc., depending on the physiochemical characteristics of original soil type. Straw addition at high rate caused significant increase in N_2_O emissions in both soils, while biochar amendment could decrease N_2_O emissions in the BH soil but caused converse effect in the TY soil. The abundance of *mcrA* and *pmoA* genes related to the production and consumption of CH_4_ changed in response to straw and biochar amendments well explained the variation of CH_4_ emissions among the treatments. Straw and biochar amendment induced visible community change in methanotrophs and *nosZ* gene containing denitrifier in the alkaline BH soil, but slight change in the acidic TY soil. The BH soil and the TY soil possessed distinct microbial community, and straw and biochar amendments caused differentiated effect on soil property of two soil types, which together explained the interactive effect of straw plus biochar application on CH_4_ and N_2_O emissions in two contrasting paddy soils. Our pot experiment suggested that biochar amendment could effectively mitigate CH_4_ and N_2_O emissions risks induced by straw application in the tested soil types, while its extensive application into different soil types requires appropriate estimation based on soil physicochemical and microbial properties, and the amount of straw return should be taken into consideration in term of gross GHG emissions.

## Author Contributions

Y-QW was responsible to most of the pot experiments, laboratorial works, data processing, and article writing. RB had an important contribution to gas collecting, sampling, and some laboratorial activities. HJD provided essential helps for the article writing and revision. L-YM paid great efforts on the setup of pot experiments, and contributed to the gas and soil samplings. BH contributed to the bioinformatics analysis of sequencing data. J-ZH provided essential ideas to the experimental design and article writing. L-MZ provided essential ideas to the experimental design and paper writing, guidance for the experiments, and was responsible for pot experiments setup, article writing and revising.

## Conflict of Interest Statement

The authors declare that the research was conducted in the absence of any commercial or financial relationships that could be construed as a potential conflict of interest.

## Footnotes

1 http://www.ncbi.nlm.nih.gov

2 http://fungene.cme.msu.edu/index.spr
