# Supplementary material for: Differentiated Mechanisms of Biochar Mitigating Straw-Induced Greenhouse Gas Emissions in Two Contrasting Paddy Soils
Source: Front Microbiol. 2018 Nov 13;9:2566. doi: 10.3389/fmicb.2018.02566 (PMC6243033; doi:10.3389/fmicb.2018.02566)
Supplement: Supplementary file 1 [file Data_Sheet_1.docx]

Supplementary Material

**Differentiated mechanisms of biochar mitigating straw-induced greenhouse gas emissions in two contrasting paddy soils?**

Ya-Qi Wang, Ren Bai, Hongjie Di, Liu-Ying Mo, Bing Han, Li-Mei Zhang *****, Ji-Zheng He

*** Correspondence:** Li-Mei Zhang: Zhanglm@rcees.ac.cn

# Supplementary Figures and Tables

## Supplementary Figures

**Supplementary Figure 1.** Dynamics of CH_4_ fluxes (mg CH_4_-C m^-2^ h^-1^) among five treatments in two soils over rice growing stages in 2016 (left side) and 2017 (right side). The same water regime of continuously flooding with a mid-drainage is applied, and indicated by light gray (flooded) and dark gray (drained), respectively. The rice growth stages are indicated by light green for seedling stage, pink for tillering stage and yellow for heading stage. The downwards arrow in black means topdressing date of nitrogen fertilizer. The bar represents the standard error of means (n=3). And the five treatments are differentiated from the diverse shapes (square in black for S0, scarlet circle for S1, blue circle for BS1, pink triangle for S2 and green triangle for BS2, respectively). The sub-graph magnified the CH_4_ fluxes where cannot be distinguished clearly.

**Supplementary Figure 2.** Dynamics of N_2_O fluxes (ug N_2_O-N m^-2^ h^-1^) among five treatments in two soils over rice growing stages in 2016 (a) and 2017 (b). The legend is same as Supplementary Figure 1.

-200

-100

0

100

TY_S0

TY_S1

TY_S2

TY_BS1

TY_BS2

BH_S0

BH_S1

BH_S2

BH_BS1

BH_BS2

*Eh* (mV)

**Supplementary Figure 3.** *Eh* in the subsurface determined at day 10 after straw addition.

**Supplementary Table 1.** The primer sets and thermal conditions used in the PCR amplifications.

| **Target genes** | **Primers** | **sequence (5' - 3')** | **length of fragments** | **Thermal profile** | **Reference** |
| --- | --- | --- | --- | --- | --- |
| Archaeal *amoA* | Arch-amoAF | STAATGGTCTGGCTTCTTC | 635 | 95℃ for 3min; 35 circles of (95℃ for10s, 55℃ for 30s,72℃ for 60s and plate read at 83℃ for 10s) | [[1](#_ENREF_1)] |
|  | Arch-amoAR | GCGGCATCCATCTGTATGT |  |  |  |
|  |  |  |  |  |  |
| Bacterial *amoA* | amoA-1F | GGGGTTTCTACTGGTGGT | 490 | 95℃ for 3min; 35 circles of (95℃ for10s, 55℃ for 30s,72℃ for 60s and plate read at 83℃ for 10s) | [[2](#_ENREF_2)] |
|  | amoA-2R | CCCCTCKGSAAAGCCTTCTTC |  |  |  |
|  |  |  |  |  |  |
| *nirK* | F1aCu | ATCATGGTSCTGCCGCG | 476 | 95℃ for 5min; 95℃ for 30s, 58℃ (a touchdown from 63℃ to 58℃, -1.0℃, 6 circles) for 30s, 72℃ for 1min and plate read at 83℃ for 10s (40 circles) | [[3](#_ENREF_3)] |
|  | R3Cu | GCCTCGATCAGRTTGTGGTT |  |  |  |
|  |  |  |  |  |  |
| *nirS* | cd3aF | GTSAACGTSAAGGARACSGG | 420 | 94℃ for 2min; 94℃ for 30s, 53℃ (a touchdown from 58℃ to 53℃, -1.0℃, 5 circles) for 1min, 72℃ for 30s and plate read at 83℃ for 10s (30 circles) | [[4](#_ENREF_4)] |
|  | R3cd | GASTTCGGRTGSGTCTTGA |  |  |  |
|  |  |  |  |  |  |
| *nosZ* clade I | nosZ_2R | CGCRACGGCAASAAGGTSMSSGT | 453 | 95℃ for 10min; 95℃ for 30s, 60℃ (a touchdown from 65℃ to 60℃, -1.0℃, 5 circles) for 30s, 72℃ for 1min and plate read at 83℃ for 10s (40 circles) | [[5](#_ENREF_5)] |
|  | nosZ_2F | CAKRTGCAKSGCRTGGCAGAA |  |  |  |
|  |  |  |  |  |  |
| *nosZ* clade II | nosZ-II-F | CTIGGICCIYTKCAYAC | 698 | 95°C for 2 min, 95°C for 30s, 54°C for 30s, 72°C for 40s and plate read at 83℃ for 10s (40 circles) | [6] |
|  | nosZ-II-R | GCIGARCARAAITCBGTRC |  |  |  |
|  |  |  |  |  |  |
| *mcrA* | ME1 | GCMATGCARATHGGWATGTC | 719 | 94℃ for 3min; 94℃ for 45s, 50℃ for 45s, 72℃ for 90s and plate read at 83℃ for 10s (35 circles) | [[7](#_ENREF_6)] |
|  | ME2 | TCATKGCRTAGTTDGGRTAGT |  |  |  |
|  |  |  |  |  |  |
| *pmoA* | A189f | GGNGACTGGGACTTCTGG | 491 | 94℃ for 2min; 94℃ for 45s, 53℃ (a touchdown from 58℃ to 52℃, -1.0℃, 6 circles) for 1min, 72℃ for 2min, and plate read at 83℃ for 10s (35 circles) ^a^  94℃ for 2min; 94℃ for 30s, 60℃ for 30s, 72℃ for 45s (35 circles); 72℃ for 5min ^b^ | [8] |
|  | mb661r | CCGGMGCAACGTCYTTACC |  |  |  |
|  |  |  |  |  |  |
|  |  |  |  | 94℃ for 2min; 94℃ for 30s, 60℃ for 30s, 72℃ for 45s (35 circles); 72℃ for 5min ^b^ |  |

**a. The thermal condition used in the qPCR for *pmoA* gene;** **b. The thermal condition used in the PCR for illumina sequence**

**Supplementary Table 2.** Spearman’s correlation among the soil properties, functional gene abundances, and the CH_4_ and N_2_O emissions during the rice growing season in 2016.

| Soil |  | pH | *Eh-surface* | *Eh-subsurface* | NH_4_^+^ | NO_3_^-^ | DOC | TN | TC | C/N | **Bacterial** | **archaeal** | ***nirK*** | ***nirS*** | ***nosZ*** | ***mcrA*** | ***pmoA*** | ***mcrA/pmoA*** |
| --- | --- | --- | --- | --- | --- | --- | --- | --- | --- | --- | --- | --- | --- | --- | --- | --- | --- | --- |
|  |  |  |  |  |  |  |  |  |  |  | ***amoA*** | ***amoA*** |  |  |  |  |  |  |
| TY | *Eh-*surface | 0.354* | 1 |  |  |  |  |  |  |  |  |  |  |  |  |  |  |  |
|  | *Eh-*subsurface | 0.222 | 0.757** | 1 |  |  |  |  |  |  |  |  |  |  |  |  |  |  |
|  | NH_4_^+^ | -0.12 | -0.671** | -0.658** | 1 |  |  |  |  |  |  |  |  |  |  |  |  |  |
|  | NO_3_^-^ | -0.294 | 0.218 | 0.176 | -0.268 | 1 |  |  |  |  |  |  |  |  |  |  |  |  |
|  | DOC | 0.722** | 0.125 | -0.052 | 0.184 | -0.364* | 1 |  |  |  |  |  |  |  |  |  |  |  |
|  | TN | 0.148 | -0.427** | -0.206 | 0.16 | -0.32 | 0.073 | 1 |  |  |  |  |  |  |  |  |  |  |
|  | TC | 0.488** | -0.088 | 0.137 | -0.095 | -0.291 | 0.26 | 0.720** | 1 |  |  |  |  |  |  |  |  |  |
|  | C/N | 0.541** | 0.021 | 0.226 | -0.156 | -0.259 | 0.288 | 0.567** | 0.979** | 1 |  |  |  |  |  |  |  |  |
|  | **bacterial *amoA*** | -0.061 | 0.499** | 0.483** | -0.621** | 0.24 | -0.441** | -0.034 | 0.051 | 0.067 | 1 |  |  |  |  |  |  |  |
|  | **archaeal *amoA*** | -0.069 | -0.218 | -0.067 | 0.258 | -0.098 | -0.082 | -0.034 | -0.103 | -0.118 | -0.028 | 1 |  |  |  |  |  |  |
|  | ***nirK*** | 0.133 | 0.067 | 0.078 | -0.2 | 0.144 | 0.157 | -0.016 | 0.006 | 0.007 | 0.005 | 0.168 | 1 |  |  |  |  |  |
|  | ***nirS*** | -0.427** | -0.769** | -0.655** | 0.531** | -0.045 | -0.282 | 0.301* | -0.109 | -0.214 | -0.23 | 0.17 | 0.019 | 1 |  |  |  |  |
|  | ***nosZ*** | -0.06 | 0.501** | 0.555** | -0.550** | 0.086 | -0.378* | -0.023 | 0.075 | 0.107 | 0.587** | -0.226 | -0.105 | -0.386** | 1 |  |  |  |
|  | ***mcrA*** | -0.466** | -0.767** | -0.575** | 0.475** | -0.202 | -0.314* | 0.503** | 0.146 | 0.027 | -0.128 | 0.018 | -0.046 | 0.710** | -0.099 | 1 |  |  |
|  | ***pmoA*** | -0.492** | -0.665** | -0.418** | 0.308* | -0.096 | -0.440** | 0.366* | 0.12 | 0.032 | 0.039 | 0.211 | 0.023 | 0.732** | -0.075 | 0.754** | 1 |  |
|  | ***mcrA/pmoA*** | -0.126 | -0.355* | -0.371* | 0.442** | -0.076 | 0.079 | 0.172 | -0.098 | -0.167 | -0.371* | -0.152 | -0.048 | 0.147 | -0.107 | 0.503** | -0.127 | 1 |
|  | CE (CH_4_) | 0.08 | 0.118 | 0.164 | 0.058 | -0.185 | 0.157 | -0.104 | -0.041 | -0.025 | -0.083 | -0.053 | 0.028 | -0.356* | 0.029 | 0.034 | -0.223 | 0.476** |
|  | CE(N_2_O) | 0.410** | 0.063 | 0.244 | -0.104 | -0.337* | 0.302* | 0.470** | 0.697** | 0.682** | 0.119 | -0.141 | 0.08 | -0.234 | 0.059 | 0.136 | 0.005 | 0.149 |
|  | CH_4_ flux | -0.171 | -0.408** | -0.251 | 0.449** | 0.101 | 0.014 | 0.296* | 0.074 | -0.002 | -0.197 | -0.011 | -0.094 | 0.147 | -0.03 | 0.514** | 0.209 | 0.587** |
|  | N_2_O flux | -0.141 | 0.25 | 0.359* | -0.314* | 0.207 | -0.341* | -0.116 | -0.07 | -0.052 | 0.341* | 0.142 | 0.061 | -0.131 | 0.297* | -0.065 | 0.032 | -0.161 |
|  |  |  |  |  |  |  |  |  |  |  |  |  |  |  |  |  |  |  |
| BH | *Eh-*surface | 0.694** | 1 |  |  |  |  |  |  |  |  |  |  |  |  |  |  |  |
|  | *Eh-*subsurface | 0.797** | 0.796** | 1 |  |  |  |  |  |  |  |  |  |  |  |  |  |  |
|  | NH_4_^+^ | 0.772** | 0.690** | 0.639** | 1 |  |  |  |  |  |  |  |  |  |  |  |  |  |
|  | NO_3_^-^ | 0.436** | 0.401** | 0.558** | 0.111 | 1 |  |  |  |  |  |  |  |  |  |  |  |  |
|  | DOC | -0.522** | -0.554** | -0.632** | -0.202 | -0.65** | 1 |  |  |  |  |  |  |  |  |  |  |  |
|  | TN | 0.019 | -0.171 | -0.12 | -0.072 | -0.136 | 0.094 | 1 |  |  |  |  |  |  |  |  |  |  |
|  | TC | -0.002 | -0.24 | -0.084 | -0.1 | -0.076 | 0.145 | 0.877** | 1 |  |  |  |  |  |  |  |  |  |
|  | C/N | -0.01 | -0.211 | 0.006 | -0.086 | 0.023 | 0.152 | 0.430** | 0.808** | 1 |  |  |  |  |  |  |  |  |
|  | **bacterial *amoA*** | -0.075 | -0.193 | -0.118 | -0.313* | 0.237 | -0.007 | 0.262 | 0.266 | 0.179 | 1 |  |  |  |  |  |  |  |
|  | **archaeal *amoA*** | -0.204 | -0.226 | -0.046 | -0.29 | 0.021 | 0.069 | 0.367* | 0.437** | 0.366* | 0.119 | 1 |  |  |  |  |  |  |
|  | ***nirK*** | -0.12 | -0.167 | -0.189 | 0.013 | -0.091 | 0.311* | 0.381** | 0.366* | 0.205 | 0.276 | 0.221 | 1 |  |  |  |  |  |
|  | ***nirS*** | -0.330* | -0.533** | -0.445** | -0.334* | -0.138 | 0.418** | 0.288 | 0.349* | 0.287 | 0.579** | 0.163 | 0.535** | 1 |  |  |  |  |
|  | ***nosZ*** | 0.581** | 0.367* | 0.428** | 0.466** | 0.359* | -0.198 | 0.343* | 0.302* | 0.163 | 0.370* | 0.026 | 0.437** | 0.323* | 1 |  |  |  |
|  | ***mcrA*** | -0.325* | -0.395** | -0.329* | -0.411** | -0.101 | 0.269 | 0.174 | 0.089 | -0.076 | 0.09 | 0.533** | 0.305* | 0.237 | -0.035 | 1 |  |  |
|  | ***pmoA*** | 0.152 | -0.17 | 0.012 | -0.109 | 0.368* | -0.219 | 0.370* | 0.312* | 0.112 | 0.543** | 0.288 | 0.359* | 0.222 | 0.428** | 0.368* | 1 |  |
|  | ***ratio*** | 0.075 | -0.049 | -0.11 | 0.239 | -0.360* | 0.175 | -0.09 | -0.18 | -0.235 | -0.374* | -0.166 | -0.065 | 0.011 | -0.132 | 0.117 | -0.314* | 1 |
|  | CE (CH_4_) | 0.051 | -0.216 | -0.05 | 0.053 | -0.118 | 0.041 | 0.077 | -0.037 | -0.182 | -0.149 | 0.286 | 0.22 | 0.043 | 0.006 | 0.466** | 0.172 | 0.299* |
|  | CE(N_2_O) | 0.536** | 0.288 | 0.347* | 0.580** | 0.15 | -0.232 | -0.033 | -0.155 | -0.249 | -0.195 | -0.156 | -0.141 | -0.129 | 0.288 | -0.046 | 0.098 | 0.424** |
|  | CH_4_ flux | -0.536** | -0.494** | -0.449** | -0.525** | -0.265 | 0.448** | 0 | -0.019 | -0.048 | 0.095 | 0.355* | 0.222 | 0.24 | -0.242 | 0.730** | 0.087 | 0.084 |
|  | N_2_O flux | -0.005 | -0.137 | 0.117 | -0.189 | 0.300* | -0.192 | 0.187 | 0.234 | 0.165 | 0.22 | 0.236 | 0.17 | 0.134 | 0.04 | 0.267 | 0.537** | -0.316* |

Abundance of each gene is in black bold.

CE means the cumulative emission of GHGs.

**Supplementary Table 3.** Alpha diversity of methanotrophs and *nosZ*-containing denitrifiers in two paddy soils.

| Stage | Soil | Treatment | *pmoA* | | | |  | *nosZ* | | | |
| --- | --- | --- | --- | --- | --- | --- | --- | --- | --- | --- | --- |
|  |  |  | ace | simpson | shannon | chao |  | ace | simpson | shannon | chao |
| Seedling | TY | S0 | 130.1 | 0.048 | 3.565 | 131.6 |  | 393.2 | 0.028 | 4.674 | 421.2 |
| (18d) |  | S1 | 140.3 | 0.051 | 3.634 | 140.1 |  | 420.5 | 0.024 | 4.800 | 428.3 |
|  |  | S2 | 130.2 | 0.052 | 3.598 | 130.4 |  | 400.9 | 0.022 | 4.823 | 411.0 |
|  |  | BS1 | 142.9 | 0.049 | 3.611 | 153.8 |  | 463.4 | 0.025 | 4.785 | 537.3 |
|  |  | BS2 | 165.0 | 0.054 | 3.650 | 164.3 |  | 491.8 | 0.023 | 4.819 | 530.3 |
|  | BH | S0 | 139.4 | 0.077 | 3.271 | 138.6 |  | 511.9 | 0.013 | 5.082 | 550.1 |
|  |  | S1 | 143.6 | 0.070 | 3.312 | 145.4 |  | 590.0 | 0.010 | 5.243 | 603.2 |
|  |  | S2 | 144.3 | 0.061 | 3.485 | 144.7 |  | 552.3 | 0.026 | 5.010 | 584.4 |
|  |  | BS1 | 102.4 | 0.079 | 3.183 | 103.3 |  | 542.6 | 0.008 | 5.360 | 530.2 |
|  |  | BS2 | 135.4 | 0.076 | 3.338 | 136.3 |  | 635.3 | 0.014 | 5.167 | 655.4 |
| Tillering | TY | S0 |  |  |  |  |  | 422.2 | 0.026 | 4.667 | 442.9 |
| (58d) |  | S2 |  |  |  |  |  | 442.1 | 0.024 | 4.758 | 473.2 |
|  |  | BS2 |  |  |  |  |  | 494.2 | 0.024 | 4.795 | 514.5 |
|  | BH | S0 |  |  |  |  |  | 540.3 | 0.020 | 4.851 | 589.0 |
|  |  | S2 |  |  |  |  |  | 624.4 | 0.010 | 5.230 | 639.6 |
|  |  | BS2 |  |  |  |  |  | 863.9 | 0.014 | 5.274 | 976.0 |
| Heading | TY | S0 |  |  |  |  |  | 512.6 | 0.028 | 4.680 | 522.2 |
| (120d) |  | S2 |  |  |  |  |  | 415.4 | 0.026 | 4.689 | 423.8 |
|  |  | BS2 |  |  |  |  |  | 544.1 | 0.024 | 4.799 | 592.1 |
|  | BH | S0 |  |  |  |  |  | 457.5 | 0.014 | 5.061 | 451.5 |
|  |  | S2 |  |  |  |  |  | 443.5 | 0.026 | 4.652 | 474.2 |
|  |  | BS2 |  |  |  |  |  | 468.4 | 0.019 | 4.933 | 576.9 |
| Origin | TY |  | 129.6 | 0.083 | 3.207 | 134.0 |  | 374.2 | 0.018 | 4.734 | 374.3 |
| (0d) | BH |  | 123.8 | 0.058 | 3.420 | 126.2 |  | 441.8 | 0.018 | 4.641 | 441.7 |

**Supplementary Table 4.** The correlation analyses between soil parameters and community composition of *nosZ* gene containing denitrifiers via the envfit function.

| Soil | TY | | BH | |
| --- | --- | --- | --- | --- |
| Parameters | r^2^ | *Pr* | r^2^ | *Pr* |
| Moisture | 0.412 | 0.001 | 0.219 | 0.030 |
| pH | 0.167 | 0.053 | 0.400 | 0.001 |
| *Eh*-surface | 0.657 | 0.001 | 0.664 | 0.001 |
| *Eh*-sublayer | 0.487 | 0.001 | 0.455 | 0.001 |
| NH_4_^+^ | 0.158 | 0.060 | 0.675 | 0.001 |
| NO_3_^-^ | 0.042 | 0.432 | 0.009 | 0.900 |
| DOC | 0.060 | 0.379 | 0.154 | 0.074 |
| TN | 0.344 | 0.001 | 0.296 | 0.002 |
| TC | 0.153 | 0.085 | 0.333 | 0.004 |
| C/N | 0.100 | 0.202 | 0.220 | 0.016 |

r^2^ represents the decisive coefficient of environmental factors on species distribution; *Pr* indicates the significant test of correlation, significant difference is accepted when *Pr* <0.050.

***References***

1. Francis, C.A., Robert, K.J., Beman, J.M., Santoro, A.E., and Oakley, B.B. (2005). Ubiquity and diversity of ammonia-oxidizing archaea in water columns and sediments of the ocean. *Proceed. National Acad. Sci.*, 102(41), 14683-14688. doi: 10.1073/pnas.0506625102.

2. Rotthauwe, J.H., Witzel, K.P., and Liesack, W. (1997). The ammonia monooxygenase structural gene *amoA* as a functional marker: molecular fine-scale analysis of natural ammonia-oxidizing populations. *Appl. Environ. Microbiol.* 63(12), 4704-4712.

3. Hallin, S. and Lindgren, P.E. (1999). PCR detection of genes encoding nitrite reductase in denitrifying bacteria. *Appl. Environ. Microbiol.* 65(4), 1652-1657.

4. Throback, I.N., Enwall, K., Jarvis, A., and Hallin, S. (2004). Reassessing PCR primers targeting *nirS*, *nirK* and *nosZ* genes for community surveys of denitrifying bacteria with DGGE. *FEMS Microbiol. Ecol.* 49(3), 401-417. doi: 0.1016/j.femsec.2004.04.011.

5. Henry, S., Bru, D., Stres, B. Hallet, S., and Philippot, L. (2006). Quantitative detection of the *nosZ* gene, encoding nitrous oxide reductase, and comparison of the abundances of 16S rRNA, *narG*, *nirK*, and *nosZ* genes in soils. *Appl. Environ. Microbiol.* 72(8), 5181-5189.doi: 10.1128/AEM.00231-06.

6. Jones, C.M., Graf, D.R., Bru, D.,Philippot, L., and Hallin, S. (2013). The uncounted yet abundant nitrous oxide-reducing microbial community: a potential nitrous oxide sink. *ISME J*. 7(2), 417-426. doi: 10.1038/ismej.2012.125.

7. Hales, B.A., Edwards, C., Ritchie, D.A., Hall, G., Pickup, R.W., and Saunders, J.R. (1996) Isolation and identification of methanogen-specific DNA from blanket bog feat by PCR amplification and sequence analysis. *Appl. Environ. Microbiol.* 62(2), 668-675.

8. Horz, H.P., Yimga, M.T. and Liesack, W. (2001). Detection of methanotroph diversity on roots of submerged rice plants by molecular retrieval of *pmoA*, *mmoX*, *mxaF*, and 16S rRNA and ribosomal DNA, including *pmoA*-based terminal restriction fragment length polymorphism profiling. *Appl. Environ. Microbiol.* 61(9), 69-70. doi: 10.1128/aem.67.9.4177-4185.2001.
